# High-Throughput Genetic Testing in ALS: The Challenging Path of Variant Classification Considering the ACMG Guidelines

**DOI:** 10.3390/genes11101123

**Published:** 2020-09-24

**Authors:** Serena Lattante, Giuseppe Marangi, Paolo Niccolò Doronzio, Amelia Conte, Giulia Bisogni, Marcella Zollino, Mario Sabatelli

**Affiliations:** 1 Section of Genomic Medicine, Department of Life Sciences and Public Health, Faculty of Medicine and Surgery, Catholic University of the Sacred Heart, 00168 Roma, Italy; serena.lattante@unicatt.it (S.L.); paolo.doronzio@yahoo.it (P.N.D.); Marcella.Zollino@Unicatt.it (M.Z.); 2 Complex Operational Unit of Medical Genetics, Department of Laboratory and Infectious Disease Sciences, A. Gemelli University Hospital Foundation IRCCS, 00168 Roma, Italy; 3 Adult NEMO Clinical Center, Complex Operational Unit of Neurology, Department of Aging, Neurological, Orthopedic and Head-Neck Sciences, A. Gemelli University Hospital Foundation IRCCS, 00168 Roma, Italy; amelia.conte@centrocliniconemo.it (A.C.); giulia.bisogni@centrocliniconemo.it (G.B.); mario.sabatelli@unicatt.it (M.S.); 4 Section of Neurology, Department of Neuroscience, Faculty of Medicine and Surgery, Catholic University of the Sacred Heart, 00168 Roma, Italy

**Keywords:** amyotrophic lateral sclerosis, ACMG guidelines, high-throughput genetic testing, gene panel sequencing

## Abstract

The development of high-throughput sequencing technologies and screening of big patient cohorts with familial and sporadic amyotrophic lateral sclerosis (ALS) led to the identification of a significant number of genetic variants, which are sometimes difficult to interpret. The American College of Medical Genetics and Genomics (ACMG) provided guidelines to help molecular geneticists and pathologists to interpret variants found in laboratory testing. We assessed the application of the ACMG criteria to ALS-related variants, combining data from literature with our experience. We analyzed a cohort of 498 ALS patients using massive parallel sequencing of ALS-associated genes and identified 280 variants with a minor allele frequency < 1%. Examining all variants using the ACMG criteria, thus considering the type of variant, inheritance, familial segregation, and possible functional studies, we classified 20 variants as “pathogenic”. In conclusion, ALS’s genetic complexity, such as oligogenic inheritance, presence of genes acting as risk factors, and reduced penetrance, needs to be considered when interpreting variants. The goal of this work is to provide helpful suggestions to geneticists and clinicians dealing with ALS.

## 1. Introduction

In recent years, the fast and steady development of high-throughput technologies for genetic analysis dramatically changed the diagnostic approach in medical genetics and research strategies in related scientific fields. Consequently, these methods also impacted the study of amyotrophic lateral sclerosis (ALS), a neurodegenerative adult onset disease involving motor neurons in the cerebral cortex, brainstem, and spinal cord. Genetic factors play an important role in ALS pathogenesis, especially in familial amyotrophic lateral sclerosis cases (FALS), where a Mendelian dominant inheritance pattern with high penetrance is detectable. FALS represent about 10% of all cases and the genetic cause has been identified in 70% of them. Although the remaining 90% of cases occur sporadically (sporadic amyotrophic lateral sclerosis, SALS) in the general population, a genetic cause or Mendelian etiology has been identified in 11% [1]. Thanks to the advent of next-generation sequencing (NGS) technologies and to the extension of the screening to SALS cases, more than 100 genes have been associated with ALS to date, with thousands of variants.

Progressively, NGS was introduced in clinical settings and genetic testing is increasingly offered to ALS patients, with relevant psychological, social, and ethical consequences, which need to be considered. Hence, genetic counseling has become an essential step to manage ALS patients, not only in familial, but also in sporadic cases [2].

An important issue raised by the availability of high-throughput genetic analyses is the interpretation of the variant consequences and, from a clinical point of view, the need for a correct variant classification in either of the two main categories: Pathogenic or benign.

To that purpose, international research groups attempted to reach consensus about criteria, which should be used for classification. The guidelines proposed by the American College of Medical Genetics and Genomics (ACMG) [3] are, undoubtedly, the most universally used for Mendelian disorders. By applying these criteria, variants should be classified in five different categories: “Pathogenic”, “likely pathogenic”, “uncertain significance”, “likely benign”, and “benign”. Recently, researchers used the ACMG standards to interpret variants found in ALS patients [4,5,6]. In this study, we discuss the application of ACMG guidelines to ALS, combining data reported in literature with results obtained from a large ALS cohort, screened with gene panel sequencing over a four-year period. The ACMG criteria are summarized in Table 1.

## 2. Patients and Methods: A Brief Description of the Studies Performed in Our Patient Cohort

### 2.1. Patients

We included in this report data of 498 consecutive ALS patients admitted to our ALS Center of the NEMO Clinical Center from September 2014 to June 2018. Patients were of Italian ethnicity, with the exception of 10 cases, and were diagnosed as possible, probable, or definite ALS according to El Escorial criteria [7,8]. Four hundred and fifty-six patients were classified as sporadic cases, while 42 patients had at least an affected relative. All the participants signed a written informed consent to participate in the study, which included gene testing, and results were communicated with genetic and psychological counseling. The study was approved by the ethics committee of the Università Cattolica del Sacro Cuore (Roma, Italy) on 21 February 2013, Prot nr. (A.133)/CE/2013.

### 2.2. Methods

Genomic DNA was extracted from peripheral blood lymphocytes using Wizard Genomic DNA Purification Kit (Promega, Madison, WI, USA).

Massive parallel sequencing of target genes was performed using the HaloPlex Target Enrichment kit (Agilent Technologies, Santa Clara, CA, USA) for library preparation. At the time of gene testing, a version specifically adapted for Ion Torrent sequencing platforms was commercially available, which went, more recently, out of production.

Custom gene panels were designed with the Agilent Sure Design software (freely available online: earray.chem.agilent.com/suredesign/). All coding exons of the genes of interest (considering the RefSeq, Ensembl, Genecode, and Vega databases) were included, with 10 bp exon padding. All the 5′UTRs and most of the 3′UTRs were analyzed, but the results are not presented here. Three different versions of the gene panel were used:

- The first version included the following genes: *ANG*, *ATXN2*, *CHCHD10*, *CHMP2B*, *CHRNA4*, *DAO*, *DCTN1*, *EPHA4*, *EWSR1*, *FIG4*, *FUS*, *HNRNPA1*, *HNRNPA2B1*, *MATR3*, *OPTN*, *PFN1*, *SETX*, *SIGMAR1*, *SOD1*, *SQSTM1*, *TAF15*, *TARDBP*, *UBQLN2*, *VAPB*, and *VCP*;
- In the second version *C9orf72*, *GLE1*, *GRN*, *MAPT*, *NIPA1*, *SS18L1*, *TBK1*, and *TUBA4A* were added;
- In the third version the following genes were further added: *ALS2*, *ANXA11*, *ARPP21*, *CCNF*, *NEK1*, and *SPG11*.

The first version was used in 51 samples, the second in 209, and the third in 238.

Barcoded libraries were sequenced on Ion Torrent sequencing platforms (i.e., on either the Ion PGM or the Ion Proton machine) (Thermo Fisher Scientific, Waltham, MA, USA), using standard kits and following the manufacturer’s instructions. Base calling, pre-processing of the reads, and short read alignment were performed with the Torrent Suite software (Thermo Fisher Scientific, Waltham, MA, USA), while variant calling was performed on the online version of the Ion Reporter software (Thermo Fisher Scientific, Waltham, MA, USA) with custom workflows.

Variant annotation was mainly performed with Annovar [9] considering the following databases, specific for the human genome assembly version GRCh37/hg19: RefSeq, UCSC Known Genes, and Gencode v31 Basic for functional gene-base annotation; gnomAD v2.1 (gnomad.broadinstitute.org) for minor allele frequency (MAF) evaluation, which used data from all populations collectively and from the European non-Finnish population, as well as from both exomes and genomes; dbscSNV [10] for splice-site variant prediction; dbNSFP v3.5 [11,12] for bioinformatic prediction of single nucleotide substitution consequences.

The InterVar software [13] was used to automatically classify variants in accordance with the ACMG guidelines. Manual curation and adjustment to the InterVar output was also performed.

All selected variants were confirmed by Sanger sequencing.

All patients were screened for the *C9orf72* pathogenic expansion by Repeat Primed PCR [14] and for the intermediate length alleles in *ATXN1* and *ATXN2* by fluorescent PCR, as previously described [15,16].

### 2.3. Result Summary

We found the *C9orf72* repeat expansion in 39 cases (19 FALS).

We identified 280 sequence variants, affecting canonical transcripts of target genes, with a MAF < 0.01 (in all the four populations considered from gnomAD 2.1; i.e., exomes of all populations, genomes of all populations, exomes of the non-Finnish European population, and genomes of the non-Finnish European population).

We detected one or more variants (including the *C9orf72* repeat expansion) in 291 patients, of which there were 82 patients with two variants, 18 with three, 4 with four, and 1 with five.

In the Supplementary Table S1, all 280 sequence variants are listed, with a further 20 variants in non-canonical transcripts and 6 variants with a MAF smaller than 0.05 in all four considered populations, but larger than 0.01 in one or two populations.

All but 3 sequence variants—*GLE1* (c.575A > G), *SETX* (c.7727T > C), and *SOD1* (c.272A > C)—in 3 different patients were heterozygous.

**Figure 1:**
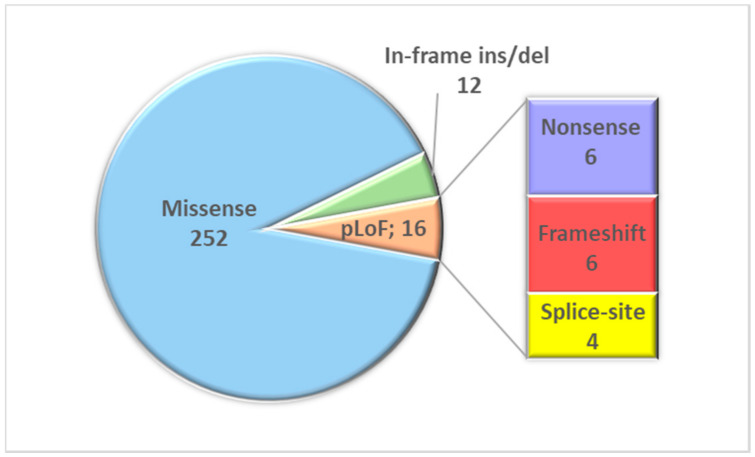
Distribution of the different variant types identified by gene panel sequencing (pLoF: Predicted loss-of-function).

We detected 6 nonsense, 6 frameshift, and 4 splice-site variants, 12 short in-frame deletions/insertions, while all the remaining sequence variants were missense (Figure 1).

**Figure 2:**
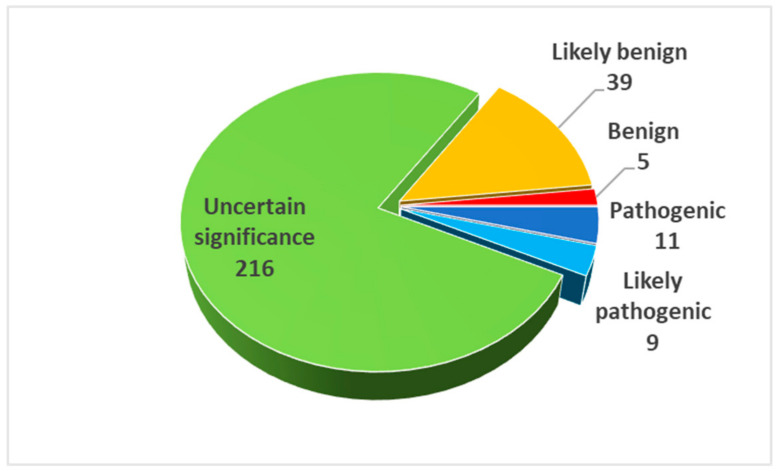
Pie chart of identified sequence variants distributed by ACMG classification, with standard InterVar interpretation of the PP2 and BS1 criteria.

Variant classification based on the results of the InterVar analysis is reported, but some variants were re-evaluated by considering further evidences not yet available for the program. For example, some variants have already been reported as responsible for ALS (PP5 evidence, often with increased strength) and the involvement of a mutational hotspot (PM1) was not correctly identified by InterVar. Manual re-evaluation of ACMG criteria is reported in brackets next to the InterVar classification. A summary of the numbers pertaining to the different classes of variants is depicted in Figure 2.

We classified 20 variants as pathogenic or likely pathogenic (Figure 2): 10 *SOD1* variants were found in 14 patients (though the heterozygous c.272A > C, detected in 1 patient, is less likely to be causative), 3 *TARDBP* variants in 8 patients, 3 *TBK1* variants in 3 patients, 1 variant in *FUS*, *NEK1*, *OPTN*, and *VCP* in 4 different patients. Nine patients with pathogenic or likely pathogenic variants had at least 1 affected relative.

In Table 2, the “pathogenic” (P) or “likely pathogenic” (LP) variants are listed, in accordance with the ACMG guidelines, with the corresponding number of patients in which they were found.

**Figure 3:**
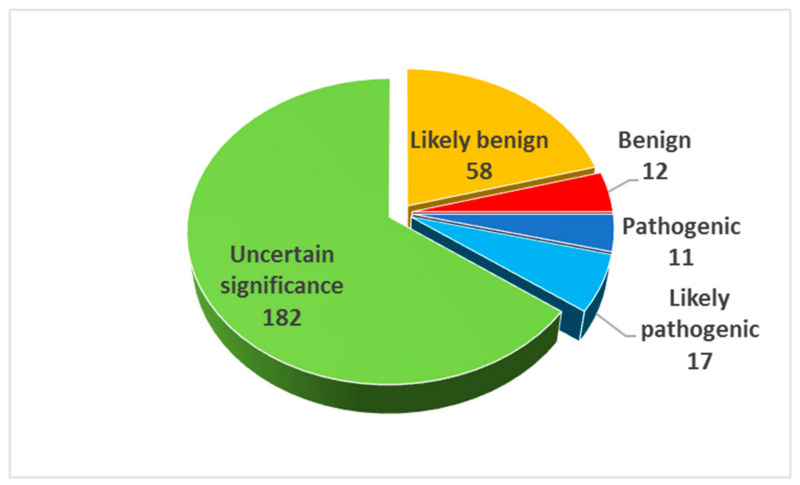
Pie chart of identified sequence variants distributed by ACMG classification, with the proposed interpretation of the PP2 and BS1 criteria.

We also tentatively assigned the BS1 label to all variants reported with a MAF > 1/10,000 in gnomAD populations, and the PP2 to all variants in genes for which pathogenic missense variants have already been reported in ALS patients (see below for the explanation). We reclassified 26 variants of uncertain significance (VUS) into “benign” or “likely benign”, and 8 more VUS into “likely pathogenic” (3 in *OPTN*, 2 in *DCTN1*, 2 in *SQSTM1*, and 1 in *DAO*) (see Figure 3 for the resulting distribution).

**Figure 4:**
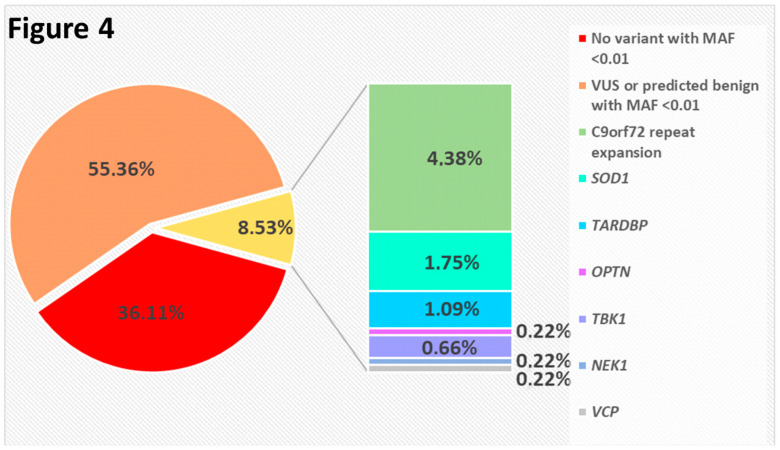
Pie chart showing the distribution of sporadic ALS cases in which we identified either a pathogenic variant in one of the indicated genes, or at least one sequence variant with MAF < 0.01 not classified as pathogenic or likely pathogenic, or no variant with MAF 0.01 at all. (VUS: Variant of uncertain significance).

**Figure 5:**
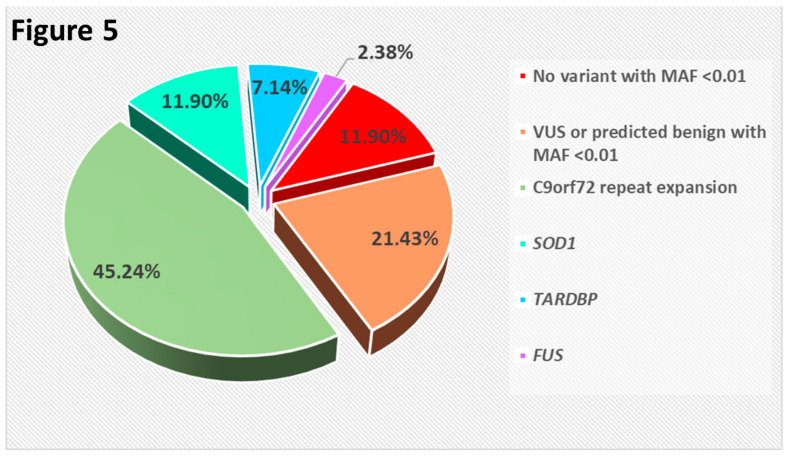
Pie chart showing the distribution of familial ALS cases in which we identified either a pathogenic variant in one of the indicated genes, or at least one sequence variant with MAF < 0.01 not classified as pathogenic or likely pathogenic, or no variant with MAF < 0.01 at all. (VUS: Variant of uncertain significance).

We identified an ALS-causative variant (including the *C9orf72* repeat expansion) in the 8.3% of sporadic ALS cases (38/456) (Figure 4) and in 66.7% of familial cases (28/42) (Figure 5). No patient had two or more variants classified as pathogenic or likely pathogenic.

## 3. ALS Genes: What Are They?

The first element, which should be considered when applying the proposed ACMG criteria to interpret sequence variants, is whether genes under consideration have been proven to be causative for the phenotype. Hence, for the purpose of clinical genetic testing, it is crucial to know the genes responsible for ALS to define those to be tested.

Unfortunately, a comprehensive list of ALS-associated genes is hard to provide. Large differences can be encountered among results of different studies. Currently, 126 genes are listed in the ALSoD database (alsod.ac.uk), but many of them likely sound unfamiliar even for most experts in the field; various possible explanations should be considered:

- Pathogenic variants in certain genes can be identified only in a very small percentage of ALS patients and, for that reason, after papers initially proposed new candidates, additional confirmatory reports are still awaited. This holds even more true for the large number of genes recently identified, since time was insufficient to gather further independent evidences [17];
- Some genes were declared ALS-related as a result of large genome-wide association studies (GWAS). These studies contributed to identify some genes relevant for ALS, such as *C9orf72* [14,18], *KIF5A* [19], and *C21orf2* [20]. On the other hand, in remaining susceptibility loci, pathogenic variants have not been reported yet [21]. Nonetheless, the genotyping of the tagging SNPs identified (which are quite common in the general population) might play an important role in the near future in clinical trials and prognostic counseling [22];
- Another category of genes and loci efficiently detected by association studies are those acting as ALS phenotype modifiers, showing in a few cases an overlap with Mendelian genes and susceptibility genetic factors [23]. At present, the mutational screening of “modifier” genes would have little or no impact in a diagnostic setting;
- A final group of genes, often listed among those someway related to ALS, is constituted by genes coding for certain proteins, which play important roles in ALS pathogenesis, but in which no causative variant has been previously identified.

Furthermore, most of the ALS genes are pleiotropic and can be associated with different clinical conditions. Frontotemporal dementia (FTD) belongs to the same phenotypic spectrum [24] and several ALS genes are involved in FTD [25]. Variants in a group of genes including *VCP*, *HNRNPA1*, *HNRNPA2B1*, and *SQSTM1* underlie the “inclusion body myopathy with early-onset Paget disease and frontotemporal dementia”, or “multisystem proteinopathy”, a heterogeneous disease, characterized by a marked variable expressivity and the involvement of different tissues [26]. Several genes are responsible for other neurological or neuromuscular disorders, such as Charcot–Marie–Tooth disease, spinal muscular atrophy, spastic paraplegia, or spinocerebellar ataxia. Other genes are instead responsible for apparently unrelated conditions, such as the open angle glaucoma [27] and short-rib thoracic dysplasia [28]. Consequently, variants classified as “pathogenic” for a certain condition should not be automatically considered causative for another, but further evidences need to be evaluated.

Table 3 provides a list of genes in which variants considered causative for ALS have been found, mainly with an assumed Mendelian inheritance. A more comprehensive list of ALS-related genes, subdivided in the previously considered categories (e.g., Mendelian, susceptibility, modifier, and functionally involved) can be found in the Supplementary Table S2.

A recent review surveyed published studies in which massive parallel sequencing methods have been used to detect ALS-associated variants, while considering Sanger sequencing as a reference [29]. Significant differences can be noticed among the various studies in terms of genes selected for screening. In some cases, selection may consider specific circumstances and aims related to research rather than diagnostic purposes.

In our study, for instance, we included genes like *C9orf72* and *ATXN2* in the sequencing panel, to evaluate the possible impact of sequence variants other than the well-known repeat expansions; indeed, for their detection, sequencing analyses are not the methods of choice. We also included *EPHA4*, for which only one functional involvement in ALS has been described recently [30], *GRN* and *MAPT*, which are both more likely frontotemporal dementia (FTD)-related genes.

**Table 3:** List of major ALS genes.

Gene	ALS *Locus*	Chr	OMIM	Inherit	oe_Mis Upper	oe_Lof Upper	pLI	Other Associated Phenotypes	Ref.
*SOD1*	ALS1	21	147450	AD,(AR)	0.918	0.978	0.17	-	[31]
*ALS2*	ALS2	2	606352	AR	0.871	0.516	<0.01	Infantile onset ascending spastic paralysis	[32]
*SETX*	ALS4	9	608465	AD	1.054	0.296	0.95	Spinocerebellar ataxia	[33]
*SPG11*	ALS5	15	610844	AR	1.163	0.812	<0.01	Charcot–Marie–Tooth disease axonal type 2X, spastic paraplegia 11 (AR), juvenile amyotrophic lateral sclerosis	[34,35]
*FUS*	ALS6	16	137070	AD,(AR)	0.737	0.237	0.99	Hereditary essential tremor	[36,37]
*VAPB*	ALS8	20	605704	AD	0.882	1.135	0.31	Spinal muscular atrophy, late-onset	[38]
*ANG*	ALS9	14	105850	AD	1.172	1.893	0.28	-	[39]
*TARDBP*	ALS10	1	605078	AD	0.385	0.275	0.98	FTD	[40,41]
*FIG4*	ALS11	6	609390	AD	0.831	1.24	<0.01	Charcot–Marie–Tooth disease, type 4J(AR); Yunis–Varon syndrome (AR)	[42]
*OPTN*	ALS12	10	602432	AD,(AR)	0.994	1.256	<0.01	Open angle glaucoma	[43]
*VCP*	ALS14	9	601023	AD	0.326	0.127	0.99	FTD, inclusion body myopathy, Paget’s disease, Charcot–Marie–Tooth disease, type 2Y Charcot–Marie–Tooth disease, type 2Y	[44]
*UBQLN2*	ALS15	X	300264	XL	0.824	0.448	0.84	-	[45]
*SIGMAR1*	ALS16	9	601978	AR	0.769	0.736	0.16	Juvenile amyotrophic lateral sclerosis, distal hereditary motor neuropathies	[46]
*CHMP2B*	ALS17	3	609512	AD	1.072	1.216	0	FTD	[47]
*PFN1*	ALS18	17	176610	AD	0.54	0.686	0.73	-	[48]
*ERBB4*	ALS19	2	600543	AD	0.805	0.216	0.99	-	[49]
*HNRNPA1*	ALS20	12	164017	AD	0.461	0.364	0.93	FTD, inclusion body myopathy, Paget’s disease	[50]
*HNRNPA2B1*		7	600124	AD	0.473	0.218	0.99	FTD, inclusion body myopathy, Paget’s disease	[50]
*MATR3*	ALS21	5	164015	AD	0.704	0.079	1	Distal myopathy with vocal cord and pharyngeal weakness	[51]
*TUBA4A*	ALS22	2	191110	AD	0.51	0.62	0.15	-	[52]
*ANXA11*	ALS23	10	602572	AD	1.143	0.98	<0.01	-	[53]
*NEK1*	ALS24	4	604588	AD	0.948	0.864	<0.01	Short-rib thoracic dysplasia 6 with or without polydactyly (AR)	[54,55]
*KIF5A*	ALS25	12	602821	AD	0.652	0.252	0.99	Spastic paraplegia 10 (AD), neonatal intractable myoclonus,	[19,56]
*C9orf72*	FTDALS1	9	614260	AD	1.065	1.468	<0.01	FTD	[14,18]
*CHCHD10*	FTDALS2	22	615903	AD	0.939	1.903	<0.01	Spinal muscular atrophy Jokela type, isolated mitochondrial myopathy, FTD	[57]
*SQSTM1*	FTDALS3	5	601530	AD	1.359	0.745	0.01	FTD, inclusion body myopathy, Paget’s disease, neurodegeneration with ataxia, dystonia, and gaze palsy, childhood-onset	[58]
*TBK1*	FTDALS4	12	604834	AD	0.794	0.42	0.07	-	[59,60]
*CCNF*	FTDALS5	16	600227	AD	0.979	0.419	0.12	-	[61]
*ATP13A2*		1	610513	AR	0.913	0.584	<0.01	Kufor–Rakeb syndrome, spastic paraplegia 78 (AR)	[62]
*ATP7A*		X	300011	AD	0.883	0.216	0.99	Menkes disease, occipital horn syndrome, spinal muscular atrophy (X-linked 3)	[63]
*C21orf2*		21	603191	AD	1.205	1.35	<0.01	Retinal dystrophy with macular staphyloma, axial spondylometaphyseal dysplasia	[20]
*CACNA1A*		19	601011	AD	0.609	0.134	1	Early infantile epileptic encephalopathy, episodic ataxia, migraine familial hemiplegic, spinocerebellar ataxia 6	[64,65]
*CYLD*		16	605018	AD	0.612	0.204	0.99	FTD, Familial cylindromatosis, Brooke–Spiegler syndrome, trichoepithelioma	[66]
*DAO*		12	124050	AD	1.102	1.697	<0.01	Schizophrenia	[67]
*DCTN1*		2	601143	AD	0.967	0.364	0.08	Perry syndrome (AD), neuronopathy, distal hereditary motor, type VIIB (AD)	[68]
*DNAJC7*		17	601964	AD	0.621	0.28	0.99	-	[69]
*ERLIN1*		10	611604	AR	0.635	0.473	0.64	Spastic paraplegia 62 AR	[70]
*ERLIN2*		8	611605	AR, AD	0.709	0.833	<0.01	Spastic paraplegia 18 (AR)	[71]
*EWSR1*		22	133450		0.718	0.278	0.99	Ewing sarcoma	[72]
*GARS*		7	600287	AD	0.911	0.402	0.3	Distal hereditary motor neuronopathy	[73]
*GLE1*		9	603371	AD	0.967	0.752	<0.01	Lethal congenital contracture syndrome 1 (AR); lethal arthrogryposis with anterior horn cell disease (AR)	[74]
*GLT8D1*		3	618355	AD	0.993	1.134	<0.01	-	[75]
*GRN*		17	138945	AD	1.049	0.483	0.06	FTD, neuronal ceroid lipofuscinosis, primary progressive aphasia	[76]
*MAPT*		17	157140	AD	0.873	0.596	<0.01	FTD, Pick’s disease, progressive supranuclear palsy	[77]
*NEFH*		22	162230		1.008	1.064	<0.01	Charcot–Marie–Tooth disease, axonal type 2CC	[78,79]
*PRPH*		12	170710	AD	1.006	1.383	<0.01	-	[80]
*RAPGEF2*		4	609530	AD	0.693	0.197	1	-	[81]
*SPAST*		2	604277	AD	0.896	0.224	0.99	Spastic paraplegia 4 (AD)	[82]
*SPG7*		16	602783	AD	1.196	1.651	<0.01	Spastic paraplegia 7 (AR)	[83]
*SS18L1*		20	606472	AD	0.818	0.306	0.98	-	[84]
*TAF15*		17	601574	AD	0.9	0.399	0.15	Chondrosarcoma, extraskeletal myxoid	[85]
*TIA1*		2	603518	AD	0.694	0.443	0.26	Welander distal myopathy	[86]

The columns entitled “oe_Mis Upper”, “oe_Lof Upper”, and “pLI” include some constraint metrics quantified by the gnomAD group in a recent paper [87]. oe_Mis Upper: Upper bound of 90% confidence interval for observed/expected ratio for missense variants. Lower values indicate more constrained genes. oe_Lof_Upper: LOEUF: Upper bound of 90% confidence interval for o/e ratio for pLoF (predicted loss-of-function) variants. Lower values indicate more constrained genes. pLI: Probability of loss-of-function intolerance; probability that transcript falls into distribution of haploinsufficient genes. Values can go from 0 (the gene is not loss-of-function intolerant) to 1 (the maximum likelihood that the gene is loss-of-function intolerant).

## 4. Applying the ACMG Standards and Guidelines for the Interpretation of ALS-Related Variants

### 4.1. The Role of Null Variants

Among the ACMG criteria, the identification of variant types causing a loss-of-function allele is the one with the greatest impact (PVS1). Frameshift, nonsense, and canonical splice-site variants—but also variants altering the start codon and deletions of the whole gene or one or more exons—are all reasonably expected to have the same effect and result in the complete absence of the protein coded by the mutated allele (or at least the complete loss of its function), with only few exceptions [3].

The main caveat to use this parameter implies that the loss of function is an ascertained mechanism of pathogenicity for the specific gene.

Considering to date available evidences, only few ALS-associate genes comply with the above specification.

The *TBK1* haploinsufficiency has been claimed to be responsible for ALS (but also FTD) since the very first studies [59,60]. More than one hundred variants have already been reported in ALS patients, with almost half of them either frameshift or nonsense [88], leading to a truncated or an absent protein product. Those variants account for about 1% in ALS/FTD patients and seem to be associated with a significantly increased risk for ALS/FTD spectrum disorders [88]. On the other hand, missense variants can be found in a further 1.8% of patients and are associated with a moderately increased ALS/FTD susceptibility [88]. Missense variants may have different consequences for protein function, ranging from alterations resembling those observed in the case of null variants (i.e., the reduction of the active phosphorylated form of the protein and of the binding and phosphorylation of its normal targets) [89], to negligible abnormalities [90]. Thus, frameshift and nonsense variants can be more easily classified as causative compared with missense, for which further evaluation, on a case-by-case basis, is required.

Optineurin and p62 are functionally related to TBK1 and are coded by the *OPTN* and *SQSTM1* genes. Variants in *OPTN* have been found in 0.5% of FALS and 0.4% of SALS patients of European origin, as well as 2.5% of FALS and 0.7% of SALS patients of Asian origin [91]. More than 60 ALS-associated variants in *OPTN* have been reported to date, with around two-fifths belonging to the protein-truncating variant (PTV) category. Furthermore, homozygous or compound heterozygous *OPTN* variants responsible for ALS were consistently truncating [91].

*SQSTM1* variants claimed to be associated with ALS so far were almost invariably missense [58,92,93]. On the other hand, heterozygous PTVs in *SQSTM1* can be responsible for Paget’s disease of bone [94] and biallelic PTVs have been identified as a rare cause for childhood-onset progressive ataxia with vertical gaze palsy [95].

Similarly to what has been previously demonstrated for *TBK1*, a statistically significant excess of loss-of-function variants between large ALS cohorts of patients and controls has been observed for *NEK1* [54,55], while an enrichment of variants affecting the splicing of exon 27 has been described for the *KIF5A* gene [19,56]. Notably, loss-of-function variants in *NEK1* are additionally responsible for “short-rib thoracic dysplasia 6 with or without polydactyly” (OMIM#263520), which is a completely different condition, showing either an autosomal recessive or a digenic inheritance [28]. On the other hand, missense variants located in the N-terminal motor domain of *KIF5A* are a known cause of spastic paraplegia type 10 (OMIM#604187) or of Charcot–Marie–Tooth disease type 2 [19,56], while de novo frameshift variants close to the 3′ end of the coding region lead to the synthesis of an elongated protein and have been associated with a congenital severe disease characterized by myoclonic seizures and progressive leukoencephalopathy [19,96,97].

The role of *SOD1* in ALS has been extensively investigated; however, variants’ molecular mechanisms leading to the disease are still unclear. Multiple lines of evidences converge to a sort of toxic gain-of-function effect, but through a variety of different biological effects [98]. Despite several studies suggesting that SOD1 loss of function might play a role in ALS [99], this possibility remains controversial [98]. More than 180 different variants have been identified over the 154 amino acid sequence of SOD1, and the vast majority of ALS-associated variants are missense [98]. Furthermore, almost all PTVs reported in ALS patients are located in the C-terminal portion of the protein, with lower chances resulting in effectively null alleles than those occurring in the N-terminal part [99]. In a very recent study a homozygous PTV in *SOD1* (leading to the total absence of enzyme activity) has been identified as responsible for a new medical condition characterized by severe developmental delay, tetraspasticity, mild cerebellar atrophy, and hypereklepsia-like symptoms [100]. No history of ALS was reported in the patient’s family. Taken together, these elements emerge against the use of the PVS1 criterion evaluating *SOD1* variants in ALS.

Regarding *TARDBP,* ALS-causing variants usually lead to the loss of nuclear functions of TDP-43 due to sequestration in cytoplasmic aggregates and compromise the TDP-43 ability supporting the splicing of specific transcripts [101]. Thus, an at least partial loss of TDP-43 function is expected in mutated ALS patients. Nevertheless, ALS-causing PTVs have been quite exceptional findings [102,103] and it still needs to be demonstrated that null alleles are pathogenic in ALS.

*FUS* variants, found in ALS patients, are commonly missense, but, in a small percentage of cases, canonical splice site substitutions or frameshift insertions and deletions have been identified [103,104]. They mainly occur in the sequence encoding for the C-terminal part of the protein, which includes the nuclear localization signal (NLS). However, it should be highlighted that these variants seem to escape mRNA degradation processes, such as nonsense-mediated decay, and lead to the production of *FUS* molecules, which are a few amino acids either shorter or longer than the normal counterpart, but in which the NLS, and sometimes adjacent regions, are replaced by a completely different peptide sequence. They have been demonstrated to affect subcellular distribution or increase the propensity to form cytoplasmic FUS-positive granules [105], with effects going beyond an expected, albeit partial, loss of function. From a clinical point of view, these frameshift variants are frequently associated with a juvenile and rapidly progressive form of ALS [106]. A specific nonsense variant, c.868C > T (commonly referred to as p.Glu290 *), has been identified as the cause of essential tremor in a large family [107]. Contrarily to ALS-associated *FUS* PTVs, the c.868C > T causes mRNA degradation through nonsense-mediated decay, suggesting that null *FUS* alleles may results in phenotypes other than ALS.

*ALS2* and *SPG11* are responsible for autosomal recessive juvenile ALS [34,108] and loss-of-function variants in these genes might be pathogenic.

In this context, the probability that a gene is “intolerant” to (putative) loss-of-function variants (pLoF) might be useful and several strategies have been proposed to provide an estimation of that [109]. In a most recent study by the gnomAD group [87], authors used their large human variant dataset to calculate a series of mutational constraint metrics describing gene-specific selection rates against pLoF variation. Some of these metrics are reported for ALS-associated genes in Table 3 (and in Supplementary Table S2). More constrained gene variants are expected to be more easily causative for diseases. However, it must be noticed that those constraints are mainly due to a process of natural selection. ALS is a late-onset disorder and at least part of ALS-associated variants would escape the selection process. This might explain why *FUS* seems to be a more constrained gene than, for instance, *SOD1* and *TBK1*, and that heterozygous-null *FUS* alleles are more detrimental in terms of genetic fitness than *FUS* PTVs found in ALS patients. In contrast, constraint metrics can be less accurate for small genes like *SOD1* [87].

In our cohort, we detected 12 nonsense or frameshift variants and 4 canonical splice-site variants (Supplementary Table S1). Five of them were classified as pathogenic (in *TBK1*, *OPTN*, and *NEK1*). Not enough evidence supports the hypothesis that the loss of function is a known mechanism of disease for ALS for the *ANG*, *CHMP2B*, *DAO*, *DCTN1*, *FIG4*, *MATR3*, *NIPA1*, and *SETX* (with 2 variants) genes. The possibility exists that at least part of the PTVs found in those genes might be pathogenic and further studies are granted. In fact, homozygous loss of function variants in *SETX* are responsible for a form of spinocerebellar ataxia with axonal neuropathy, while heterozygous PTVs in *CHMP2B* and *GRN* have already been found as causative in patients with FTD. The consequences of the *SOD1* intronic variant, adjacent to the donor splice site, are yet to be determined, because no alteration in the protein sequence might be revealed at all.

### 4.2. The Role of Missense Variants

The ACMG guidelines provide some criteria specifically pertaining to missense variants. As previously highlighted, the vast majority of genetic variants associated with ALS are missense. In our study, 251/280 (90%) variants were missense, and 15/20 (75%) of those pathogenic or likely pathogenic. Hence, the PP2 evidence is satisfied if “very little benign variation in the gene” exists. A threshold for “a very little” amount of benign variants in a single gene is hard to define. A rough idea is provided evaluating the gnomAD gene constraint metrics reported in Table 3 and Supplementary Table S2. Specifically, large differences can be noted among ALS-associated genes comparing the number of missense variants expected in a gene with those observed in general population. In general, the most relevant genes show some degree of missense depletion. Hence, the PP2 criterion should be applied by default for all major ALS genes. By contrast, the supporting evidence for benignity BP1 should be disregarded, because, in ALS genes, a number of missense variants have already been accounted for being pathogenic, apart from the *C9orf72* and other genes with repeat expansions.

We tried to assign the PP2 evidence to all missense variants, apart from those in *ATXN2*, *EPHA4*, *CHRNA4*, *EWSR1*, *ARPP21*, *C9orf72*, and *GLE1*, which would change the classification of 8 VUS into “likely pathogenic” variants, specifically in *OPTN* (3 variants), *SQSTM1* (2), *DCTN1* (2), and *DAO* (1).

A strong impact on the evaluation is given by the occurrence of the same amino acid change of a well-known causative variant (PS1). Currently, a tendency exists to consider all predicted variants resulting in the same amino acid change to have the same consequences. As adverted by the ACMG guidelines, the variant might act directly through the specific DNA change or might modify the protein sequence through other ways (e.g., by altering the mRNA splicing).

When a variant affects an amino acid already involved in a pathogenic missense variant, but causes a change into a different residue, the PM5 condition is fulfilled with a moderate impact. Several examples can be found in genes such as *SOD1*, *FUS*, and *TARDBP*, in which a large number of causative variants have already been reported. For *SOD1*, more variants than amino acids in the protein were revealed; thus, it is quite expectable that the same amino acid is involved multiple times.

While ALS-associated variants in *SOD1* are spread throughout the whole gene length, pathogenic variants in *FUS* and *TARDBP* are more frequently found in glycine-rich domains or at the level of the C-terminal nuclear localization signal (for *FUS*) [103], allowing the use of the PM1 criterion. We have already cited that *KIF5A* splice-site variants discovered in ALS patients mainly affect the splicing of exon 27. Bioinformatic tools, like InterVar, to interpret variants may help to identify mutational hotspots or well-established functional domains. However, in our patients, the InterVar annotation applied the PM1 evidence to a significant variant number, including most of the *SOD1* variants, but not to variants in *FUS* and *TARDBP* hotspot regions, thus requiring manual adjustment.

Synonymous variants are sometimes reported in the screening of large ALS cohorts, but usually with no demonstrated functional consequences or, at most, with an unreplicated statistical association [110]. Consequently, they are commonly considered benign. However, according to the ACMG guidelines, the circumstance that a variant is expected to be synonymous only guarantees a supporting evidence of benignity (BP7), and only if computational evidence predicts no impact on RNA splicing and no evolutionary conservation of the nucleotide. Therefore, the integration of splice prediction tools in the variant annotation pipeline would be a good practice for a more comprehensive analysis, as already implemented in some literature studies [5].

Indeed, in silico algorithms to predict functional consequences have often been used to evaluate variants in ALS research. Converging computational evidence from different bioinformatics tools is considered supporting evidence for either pathogenicity (PP3) or benignity (BP4). Apart from the programs specifically exploring splicing alteration, an abundance of tools has been developed to analyze missense variants, while others are suitable to predict the effect of coding and noncoding variants [111]. In many cases, tools rely on the evaluation of the evolutionary conservation, protein structure, or function (or a combination thereof).

### 4.3. In-Frame Deletions and Insertions

In the ACMG guidelines large protein length changes, due to in-frame deletions, insertions or stop loss changes, are considered moderate evidence to pathogenicity (PM4), while small in-frame deletions/insertions in repetitive or poorly conserved regions are more likely benign (BP3). In ALS genes, such variants have been rarely reported. We can cite recurring deletion or insertion of one or more codons coding for glycines in the glycine-rich region of *FUS*, which are also reported at a low frequency (MAF < 0.002) in the general population and are yet to be considered of uncertain significance. Repeat length variations constitute a specific variant category, which play a relevant role in certain, mainly neurologic, diseases. Apart from the well-known repeat expansion in the first *C9orf72* intron, intermediate-length polyglutamine expansions in *ATXN1* and *ATXN2* has been associated with an increased risk for ALS [16,112,113,114], which acts as a disease modifier in *C9orf72* expansion carriers [115], or modifier of ALS survival [116].

Among our tested patients, we found 14 patients carrying 12 different in-frame deletions (9), duplications (2) and insertions (1), with 6 of them involving the glycine-rich region of *FUS* and 1 *TBK1*, classified as a variant of uncertain significance never described in the general population.

### 4.4. De Novo Variants and Parental Testing

In congenital or early-onset diseases, with severe clinical manifestations, heterozygous de novo variants are usually considered first-line candidates, when both parents are healthy. Hence, they are accounted as strong support for pathogenicity in the ACMG guidelines (PS2 or PM6). De novo variants in ALS patients might explain at least part of the sporadic cases observed. Several de novo ALS genes variants have been observed, appearing to be a common cause of *FUS*-related early-onset ALS [117,118,119,120,121,122,123,124,125]. By searching for such kind of variants in sporadic ALS trios (i.e., in affected patients and their healthy parents) with exome sequencing, new genes were proposed as possibly related to ALS: *SS18L1* [84], *CLEC4C* [126], and *RAPGEF2* [81]. However, these genes still await further evidences to confirm their role. Furthermore, a more recent study [127] rejected the assumption that de novo variants result in a significant portion of ALS cases.

In a clinical setting, PS2 criterion evaluation is hampered by the fact that parent testing is often impossible, either because parents are already deceased, or testing has limited utility, since pathogenic ALS variants are usually inherited from healthy parents, as a consequence of a reduced penetrance. In fact, in our cohort, no healthy parent was tested, except for a trio in which we found the *C9orf72* repeat expansion in both the patient and his unaffected father.

The PM3 and BP2 criteria are closely linked to test parental samples, since they consider whether two variants observed in the same gene in a single patient occur in cis (the same copy of the gene) or in trans (different copies of the gene). They should be only applied in very few cases in which two (or more) heterozygous variants were detected in known ALS-associated genes with recessive inheritance (e.g., *ALS2*, *SPG11*, and *SIGMAR1*).

### 4.5. Variant Frequency in Cases and Control Populations

A relevant element, invariably considered in variant interpretation, is the comparison of the variant allele frequency between patient and controls. If the MAF in the general population is higher than 5%, the variant should be reasonably considered benign (BA1). It is worth recalling that the ACMG guidelines are specific for gene variants responsible for Mendelian disorders and cannot be used to infer the role of common variants associated with an increased susceptibility to the disease.

If the MAF in the control population is greater than expected for the disorder, a strong evidence for benignity (BS1) is considered. Consequently, a MAF threshold for a condition like ALS needs to be defined, in which significant confounding factors such as late-onset of the disease, reduced age-dependent penetrance (which may have different values among different variants), possible role of the oligogenic inheritance, and multifactorial origin should be considered [128]. The development and use of statistical strategies incorporating confounders are necessary. Whiffin and colleagues recently proposed “a statistical framework for the frequency-based filtering of candidate disease-causing variants, accounting for disease prevalence, genetic and allelic heterogeneity, inheritance mode, penetrance, and sampling variance in reference datasets” [129]. A companion online tool was developed (www.cardiodb.org/allelefrequencyapp/) to compute the maximum expected allele frequency of a variant considered a credible disease-causing candidate. If we attempted to calculate this value for ALS, we would need to make some assumptions about the genetic ALS not always valid:

- The inheritance model to consider is “monoallelic” for most ALS genes;
- The “prevalence” value required should not correspond to ALS prevalence but rather to the cumulative lifetime risk of ALS (i.e., ~1/300) [130], which would more closely describe the fraction of the population carrying variants responsible for ALS;
- The allelic heterogeneity is likely very low and we can conservatively set it at 0.1, assuming that for each ALS-gene there should be at least 10 different pathogenic variants (the *C9orf72* repeat expansion would be the only relevant exception, but we do not expect to find other sequence variants with such features in ALS);
- The allelic heterogeneity could also be set at 0.05, considering the amount of genes that have been claimed so far of being responsible for ALS and the fact that none of them appears to be responsible for more than 5% of cases (again, with the well-known and expectedly isolated exception of the *C9orf72*);
- The penetrance is even more difficult to evaluate, since a large difference among genes and variants may exist. A recent estimate of the *C9orf72* repeat expansion penetrance shows that it might be nearly complete at 83 years [131]. Supposing that for other genes might be smaller at the same age, we could tentatively consider a value of 80%. Nonetheless, this value should be modelled to the age distribution of reference cohorts.

With the above-mentioned values, we obtain a “maximum credible population AF” for ALS of about 1/10,000 alleles. Considering that we tried to be very conservative for most of the parameters, the threshold could be even lower. Applying this threshold to our cohort, we reclassified 28 VUS as “likely benign” (Supplementary Table S2). However, we need to be cautious in the definition and in the systematic application of this threshold, since it is based on an autosomal dominant model of inheritance, while greater frequencies need to be defined for the autosomal recessive, but also for the possible digenic or oligogenic models. In fact, a distinction based on genes cannot be performed, since genes such as *SOD1* and *FUS* may show different patterns of inheritance. Therefore, in our results, we considered a traditional threshold of 1% for the MAF to include, for example, possible recessive variants [132].

The observation of a significant difference in variant prevalence between affected individuals and controls (PS4) can provide strong evidence for pathogenicity, which, for example, led to the identification of the *C9orf72* repeat expansion [14,18]. For most of the other ALS-causative variants, the size of case-control cohorts needs to be large enough to reach indisputable statistical significance. Hence, such cohorts are quite invaluable, as is the availability of the related results. An important effort to sequence a large number of ALS patients and controls has been carried out in the “Project MiNE”, built on a worldwide collaboration between ALS centers and foundations [133]. The obtained data are publicly shared on an online database (www.projectmine.com).

Unfortunately, a great percentage of pathogenic variants can be found only in a single affected individual (or in an isolated family). Thus, even though the absence of a variant in the general population has a moderate evidence level of pathogenicity (PM2), it is often insufficient for a definitive classification (other than “uncertain significance”).

### 4.6. Familial Segregation

Investigating the segregation of a specific variant in the family could be a useful tool suggested by ACMG guidelines: The segregation of the variant with disease in affected family members could support pathogenicity (PP1), while the lack of cosegregation could be more likely associated with a benign role (BS4). Nevertheless, the segregation should be carefully evaluated: if a variant co-segregates with a disease, it will be more likely associated with its manifestation, but not necessarily pathogenic by itself.

In ALS, variants found in FALS and SALS cases should be differently assessed. FALS cases are more often caused by variants in “major genes” and affected individuals share usually the same variant.

In our cohort of 42 FALS, we confirmed the presence of variants identified in all affected relatives available for testing: 2 families carrying the *C9orf72* expansion (2 siblings and 2 cousins), one with the *TARDBP* p.G376D (a cousin and an uncle), and 2 with the *SOD1* p.L144F and p.I150T (3 siblings) variants. A few families shared the variant, presumed to be pathogenic, with some but not all affected members, leading to the hypothesis that the disease might have environmental or epigenetic causes [134].

On the contrary, in SALS cases the variant segregation could be studied in healthy subjects, but this would raise some ethical issues to be taken into serious account, both in diagnostic and research settings, and standardized protocols, ethical guidelines and pre- and post-test counseling should be followed [2]. After providing the proper counseling and obtaining subjects’ consent, we tested DNA samples from 3 healthy siblings of a patient carrying the p.G294V variant in *TARDBP*, thus identifying the variant in 2 of them. The point that the variant is carried by healthy subjects does not mean that it does not have a pathogenic role, also considering that the subjects can be healthy at the time of genetic testing, but they could develop the disease later on.

Incomplete penetrance of variants associated with ALS was indeed described for different genes. First of all, obligate carriers of *SOD1* variants, harbored by family members with ALS, did not develop any symptoms [135,136,137,138]. On the contrary, pathogenic variants in *FUS*, located in the functional C-terminal domain, showed high penetrance. The variants arose de novo in all patients tested for pathogenic *FUS* variants described in the literature [117,118,119,120,121,122,123,124,125]. An ACMG recommendation regarding the segregation studies is to include distant relatives; this strategy could increase the possibility that a shared variant is consistently associated with the phenotype. By applying exome sequencing to a large Caucasian family, the gene *MATR3*, already known to be associated with a form of distal myopathy, has been linked to ALS [51].

The importance of obtaining an accurate variant classification in FALS cases, in which a monogenic cause is more often found, might have consequences in SALS patients. Variants in familial cases can be more easily classified, based on their segregation, and hence promptly identified, should they be found in sporadic cases.

### 4.7. Functional Studies

To support genetic variants pathogenicity, the ACMG guidelines suggest validated, well-established, and reproducible functional studies to represent a powerful tool. Functional studies, performed in vitro or in vivo, can show a damaging effect on protein function, thus supporting the pathogenicity of the variant tested (PS3), or no effect, thus supporting a benign impact of the variant (BS3).

Enzymatic commercially available kits are well-established tools to test the impact of a variant, but such a test does not exist for ALS yet. Animal models are widely used in ALS research especially with the goal to investigate underlying mechanisms of disease pathogenesis. Therefore, animal models of the most common ALS pathogenic variants, not only on mice but also on monkeys and pigs, have been created to perform electrophysiological studies [139] and to test drugs at the pre-symptomatic stage [140]. All these models are expensive and time-consuming and therefore not applicable to test the pathogenicity of all novel variants. Small models, such as *Danio rerio* (zebrafish) and *Drosophila melanogaster* (common fruit fly), can be used to test more quickly the role of variants and the interactions between different genes [141,142].

In vitro studies can be performed based on the observation that variants in ALS-associated genes often cause the production of aberrant proteins, which are prone to aggregate. Commercial cell lines have been widely used, especially in the past years, and have been engineered to carry ALS-mutations. Recently, cells directly derived from patients, such as skin fibroblasts, have been used to obtain induced pluripotent stem cells (iPSCs).

Functional studies are especially needed for variants found in genes with a small number of descriptions. Cellular models have been mostly used to assess the pathogenicity of variants in novel genes. Frequently used simple assays evaluate the impact of the variant on mRNA level and on protein expression. In ALS patients, nonsense and frameshift variants in *TBK1* act through a haploinsufficiency mechanism. Cellular studies have been performed to better characterize the role of missense variants, demonstrating that missense variants affect phosphorylation of IRF3, a TBK1 target, OPTN binding and phosphorylation, as well as autophosphorylation of TBK1 [89,143]. The mechanism underlying TBK1 pathogenesis can be considered the same for nonsense and missense variants, especially when located in critical domains. Additionally, we tested *TBK1* mRNA and protein levels in fibroblasts from ALS patients carrying novel variants, to confirm that loss-of-function is a consistent mechanism [144]. Results of in vivo and in vitro experiments need to be carefully evaluated because of the indirect correlation to the phenotype observed.

### 4.8. Reputable Source

An element with increasing relevance in the next future are databases reporting identified variants with their associated phenotypes and the interpretation of their functional role. Especially for ALS, in which the vast majority of the variants identified are novel, it is very important to share variants in dedicated databases. To date, the ClinVar database (www.ncbi.nlm.nih.gov/clinvar/intro/) is likely the most renowned, “freely accessible, public archive of reports of the relationships among human variations and phenotypes, with supporting evidence”. The ALSoD database (alsod.ac.uk) specifically includes variants in ALS genes. Additionally, the Project MiNE archive might be useful to gain insights into possible pathogenic roles of certain variants, even though no inference is made about the clinical significance of the reported variants.

In the AMCG guidelines, the PP5 (for pathogenicity) and the BP6 (for benignity) criteria consider if a variant has already been classified either as pathogenic or as benign by other scientists. As a baseline, this is considered a supporting evidence, but the impact may be risen up even to “very strong”, based on the amount of different reports supporting the same interpretation with no conflict among them. For most of the missense variants in our study classified as “pathogenic” or “likely pathogenic” we could consider a stronger PP5 evidence, because those variants have already been reported multiple times in scientific literature. The PP5 and the BP6 should actually be used if the underlying evidences used for the interpretation are not available or have not been clearly specified; instead, the criteria relevant to the evidence should be used.

### 4.9. Further Considerations

Applying the ACMG guidelines to evaluate variants found in our patient cohort, we could notice that some evidences can only rarely, if ever, be used while others have a strong impact on variant classification.

Furthermore, differences may occur in the application of specific criteria: For instance, changes for the PM1, PM2, PP2, and BS1 criteria led us to reclassify about the 14% of variants (40/280). Eight more variants than those initially considered were classified “likely pathogenic”, applying more extensively the PP2 evidence. However, we do not feel as confident for them as for the variants reported in Table 2. In addition, several other VUS exist for which we might expect a higher change of being involved in ALS pathogenesis, which would be worthy to be further investigated.

Comparing our results with those presented in previous reports [5,6], we can confirm that the majority of pathogenic variants identified are *C9orf72* repeat expansions (39/59, 66%), followed by *SOD1* (10/59, 17%) and *TARDBP* (8/59, 14%) variants.

As expected, “pathogenic” or “likely pathogenic” variants can explain a large proportion of FALS cases (67%), but only 8.5% of SALS patients.

We were probably more conservative than other authors [5] in variant classification, since we found pathogenic or likely pathogenic variants only in 8 out 39 tested genes (21%). For instance, Pensato and colleagues identified these categories of variants in 12 out of 46 genes (26%) [5].

A summary of the possible considerations about the application of the specific ACMG criteria and their relevance in ALS is presented in Table 1.

Based on evidences discussed in the previous paragraphs, ALS-specific guidelines should be developed addressing the following aspects:

- The definition of an allele frequency to be considered either in favor (PM2) or against (PP2) the pathogenic role of variants. Different thresholds should be established considering the different models of inheritance;
- The study of larger cohorts of patients and controls to allow for a more precise evaluation of the relative risk associated with as many different variants as possible (PS4);
- The collaborative efforts in sharing data about patients tested and variants identified from laboratories around the world. This would lead to a more frequent use of criteria that may have a strong impact on variant classification (i.e., PS1, PM1, PM5, PP5, and BP6);
- The development and the selection of computational tools that explore the possibility of a certain variant to have ALS-specific functional consequences (PP3 and BP4);
- The identification of causative variants in families with a relevant number of affected people. Though they are a rare finding, their relevance is invaluable for both the identification of new ALS-associated genes and the classification of further variants (PP1);
- The development of standardized functional assays for the assessment of the variant involvement in ALS pathogenesis.

It is undeniable that most of the above listed steps would primarily benefit from improved knowledge of the pathogenic mechanisms underlying ALS.

## 5. The Complex Genetic Architecture of ALS and the Challenges in the Identification of Pathogenic Variants

The idea that ALS is caused by a combination of genetic and environmental factors is increasingly supported by recent genetic results [145,146]. The lack of segregation of pathogenic variants in some affected family members, the detection of pathogenic variants in healthy individuals, and the co-occurrence of variants in different ALS genes support the general idea that the genetic architecture of ALS is more complex than expected when considering only Mendelian patterns of inheritance.

To quantify the contribution of genetic factors in ALS pathogenesis, heritability was measured by different studies. Testing monozygotic and dizygotic twins, concordant and discordant for the ALS phenotype, the estimated heritability resulted to be 60% [147]. Genome-wide complex trait analyses were performed on different sets of genome-wide association studies including thousands of ALS cases and controls. All known single-nucleotide polymorphisms associated with ALS were tested simultaneously, leading to the conclusion that common genetic variants account for 8.5–21% of heritability [148,149]. Recently, a large population-based pedigree study showed that the overall heritability of ALS is up to 50% and that first-degree relatives of ALS patients have an increased risk to develop the disease, even in the absence of pathogenic variants [150]. Although a large number of genes have been correlated to ALS, there is still a “missing heritability”, which might reside in other variant categories.

On one side, some variants are expected to have a small effect size, since they cannot be causative by themselves, but they play a role in increasing the risk or the susceptibility to disease. Common variants belonging to this category should be efficiently explored by large GWAS, but rarer small-effect variants are very difficult to identify with the currently available methods [151]. Their importance should not be disregarded, since they could underlie a significant portion of ALS cases, if taken together.

On the other side, specific variant types might exist, which are not commonly detectable by the currently used analysis methods, such as repeat expansions [152,153], variants in non-coding regions [154,155], and structural variants, which have been so far poorly explored in ALS [156].

A further, major source of complexity in the genetic architecture of ALS comes from the suggested oligogenic model of inheritance, recently invoked by several studies and evidences [157,158]. It means that pathogenic variants in different genes may be needed to fully express the disease. The burden of rare variants could modify the phenotype [159], influencing the age at onset, which is anticipated in patients with multiple variants [160,161] and could have an impact on survival [162].

Another potential genetic factor not substantially addressed in ALS yet, which may be important to consider in future studies, is constituted by somatic mutations. Post-zygotic variants are not accountable in the disease heritability, but several authors hypothesized that they could play manifold roles in neurodegeneration [163,164].

Finally, the last layer of complexity in the genetic architecture of ALS is constituted by all those elements that, by definition, are not “genetic” and are collectively designated as environmental factors. Cigarette smoking [165], head trauma [166], and atypical infectious agents such as retroviruses [167] are some examples of environmental risk factors [168]. A unifying theory that considers both the contribution of genetic variants and their interplay with the “environment” has been proposed in the so-called “multi-step model”. It suggests that the pathological process underlying the disease could exist from birth, but the accumulation of variants, toxic effects of proteins, and environmental factors are needed to trigger the disease [169,170,171]. Variants in some genes, such as *SOD1* and *C9orf72*, could have a more severe effect, while multiple variants in other genes with a lower impact are needed to exceed the threshold [172,173,174,175,176].

## 6. Conclusions

Considering all aspects examined, it becomes evident that the mere application of the ACMG guidelines to interpret genetic variants in ALS cannot be considered sufficient to address various issues related with the implementation of the last-generation genetic analysis technologies in a diagnostic and clinical setting.

ALS-specific guidelines would allow a more adequate classification of the large number of variants with uncertain significance, which have been constantly discovered. This can be realized only through increased collaborations, leading to the realization of genetic researches on large cohorts of patients and controls and sharing of obtained data, from single sample analysis or from great multi-laboratory projects.

Furthermore, it is critical to develop and implement new analysis strategies that could facilitate the discovery and, moreover, the validation of genetic factors involved in the pathogenesis of ALS that are yet unknown.

Nonetheless, the most urgent issue is to define a strategy to integrate constantly increasing amounts of data for genetic studies in the patient care routine.

Genetic tests may have great impact from a medical, psychological, and social point of view. A growing number of laboratories around the world offer genetic analyses to ALS patients and their families. However, several aspects are still to be considered:

- ALS diagnosis remains based on clinical, not genetic criteria. The improvement of the currently available variant classification systems might provide better foundations to use genetic results among the diagnostic criteria. This will acquire increasing importance if a presymptomatic diagnosis should become crucial in therapeutic interventions yet to come;
- Patient and family counseling is already a significant step in the process of care, yet consensus guidelines are missing, which could give indications of the results worth being communicated as useless or, even worse, a source of anxiety for patients and their relatives;
- Though the role of small-effect variants may appear of limited interest, their identification should not be neglected, since they could constitute a potential target for possible tailored therapeutic approaches in the context of the personalized medicine opportunity.

At this point, it appears evident that any further progress to understand and define the precise effect of the various genetic variants on the ALS phenotype needs international collaborative efforts and the development of specific consensus guidelines.

## Figures and Tables

**Table 1:** List of the American College of Medical Genetics and Genomics (ACMG) criteria, with their acronyms, relevance in amyotrophic lateral sclerosis (ALS), and considerations about their application in ALS variant interpretation.

Code	Criteria	Level of Evidence	Relevance in ALS	Considerations for Their Application in ALS
PVS1	Null variant (nonsense, frameshift, canonical splice sites, etc.) in a gene for which loss of function is a common mechanism of disease	Very strong	++	So far, applicable for variants in following genes (in ALS): *ALS2*, *SPG11*, *TBK1*, *OPTN*
PS1	Same amino acid change as a previously established variant	Strong	++	Frequently applicable for genes more extensively investigated than others, e.g., *SOD1*, *FUS*, and *TARDBP*
PS2	De novo variant in a patient with the disease and no family history	Strong	+	Parental testing rarely performed on routine basis. To be assessed mainly in early-onset cases (e.g., *FUS*-related ALS)
PS3	Well-established functional studies supportive of a damaging effect on the gene or gene product	Strong	+	Functional studies already available for a large series of variants, but well-established tools are still missing for routine assessment of variant role
PS4	The prevalence of the variant in affected individuals is significantly increased compared with the prevalence in controls	Strong	++	Data from large cohorts of patients and controls are already publicly available. Larger studies are still necessary to reach statistical significance for as many variants as possible
PM1	Located in a mutational hotspot and/or critical and well-established functional domain without benign variation	Moderate	++	Easy to apply for variants in genes such as *FUS*, *TARDBP*, and *KIF5A*. Further studies needed for an extension of the list
PM2	Absent from population controls	Moderate	+	To be considered in any case. Consensus is missing about allele frequency thresholds (i.e., variants responsible for ALS can be found in general population at low frequencies)
PM3	For recessive disorders, detected in trans with a pathogenic variant	Moderate	+	To be considered when variants in genes responsible for recessive forms of ALS are found
PM4	Protein length changes as a result of in-frame deletions/insertions in a non-repeat region or stop-loss variant	Moderate	+	A small subset of variants found to date belong to this category, and their role is still not well-defined
PM5	Novel missense at an amino acid residue where a different pathogenic missense change has been seen before	Moderate	++	Frequently applicable for genes more extensively investigated, e.g., *SOD1*, *FUS*, and *TARDBP*
PM6	Assumed de novo, without confirmation of paternity and/or maternity	Moderate	+	Parental testing rarely performed on routine basis. To be assessed mainly in early-onset cases (e.g., *FUS*-related ALS)
PP1	Cosegregation with disease in multiple affected family members in a gene definitively known to cause the disease	Supporting*	++	To be used for familial cases. Families with a larger number of affected members would allow for increased significance
PP2	Missense variant in a gene that has a low rate of benign missense variation and in which missense variants are a common mechanism of disease	Supporting	++	Fulfilled for most ALS genes
PP3	Multiple lines of computational evidence support a deleterious effect on the gene or gene product	Supporting	++	Routinely used for variant evaluation in ALS
PP4	Patient’s phenotype or family history is highly specific for a disease with a single genetic etiology	Supporting	+	Applicable only in specific situations (i.e., large families with apparently Mendelian inheritance or suggestive phenotypes, such as juvenile forms)
PP5	Reputable source reports variant as pathogenic, but the evidence is not available to the laboratory to perform an independent evaluation	Supporting*	++	Evidence strength depending on number and details of reports
BA1	Allele frequency is >5% in general population	Stand-alone	++	To be considered for any variant
BS1	Allele frequency is greater than expected for disorder	Strong	++	To be considered in any case, though a consensus is missing about allele frequency thresholds
BS2	Observed in a healthy adult individual with full penetrance expected at an early age (the genotype must be consistent with the disease pattern of inheritance)	Strong	−	Not to be applied: In ALS, penetrance is not expected to be complete at an early age for any gene
BS3	Well-established functional studies show no damaging effect on protein function or splicing	Strong	+	Functional studies already available for a large series of variants, but well-established tools are still missing for routine assessment of variant role
BS4	Lack of segregation in affected members of a family	Strong	+/−	To be used in familial cases. Families with a larger number of affected members would allow for increased significance. However, reduced penetrance can explain the observation of healthy cases with the variant
BP1	Missense variant in a gene for which primarily truncating variants are known to cause disease	Supporting	−	Not to be applied: Pathogenic missense variants are commonly found in ALS genes (apart from those in which a repeat expansion is the causative one)
BP2	Observed in trans with a pathogenic variant for a fully penetrant dominant gene/disorder or observed in cis with a pathogenic variant in any inheritance pattern	Supporting	+/−	The co-occurrence of two or more pathogenic variants in ALS patients is not a rare event
BP3	In-frame deletions/insertions in a repetitive region without a known function	Supporting	−	Not to be used, since repeat expansions are a common pathogenic mechanism in ALS (and may occur in introns)
BP4	Multiple lines of computational evidence suggest no impact on gene or gene product	Supporting	++	Routinely used for variant evaluation in ALS. However, there is the need for more specific tools
BP5	Variant found in a case with an alternate molecular basis for disease	Supporting	+	Rarely useful
BP6	Reputable source reports variant as benign, but the evidence is not available to the laboratory to perform an independent evaluation	Supporting	++	Evidence strength depending on number and details of reports
BP7	A synonymous for which no splicing alteration is predicted AND the nucleotide is not highly conserved	Supporting	++	Routinely used for variant evaluation in ALS

++: Criteria that should have a more relevant impact on variant classification in ALS patients, either because they are expected to be frequently considered or because they may be crucial for a conclusive interpretation. +: Criteria that are less likely to impact on ALS variant classification. −: Criteria that should not be used in ALS variant classification. +/−: Criteria for which specific cautions should be considered. *: Criteria that may provide stronger evidence in specific cases.

**Table 2:** List of pathogenic or likely pathogenic variants identified.

Gene	Transcript	Exon	cDNA	Protein	Class	ACMG Criteria	Nr.
*TARDBP*	NM_007375	6	c.881G > T	p.G294V	P	PM1, PM2, PM5, PP2, PP5(strong)	1 ^f^
*TARDBP*	NM_007375	6	c.1127G > A	p.G376D	LP	PM1, PM2, PM5, PP2	1 ^f^
*TARDBP*	NM_007375	6	c.1144G > A	p.A382T	P	PM1, PP2, PP5 (very strong)	6 ^f^
*OPTN*	NM_021980	4	c.451C > T	p.Q151*	P	PVS1 (very strong), PM2, PP3	1
*TBK1*	NM_013254	6	c.684dupT	p.R229*	P	PVS1 (very strong), PM2, PP3	1
*TBK1*	NM_013254	13	c.1445_1446delAT	p.Y482*	P	PVS1 (very strong), PM2, PP3	1
*TBK1*	NM_013254	19	c.2040dupT	p.N681*	P	PVS1 (very strong), PM2, PP3	1
*FUS*	NM_004960	14	c.1540A > G	p.R514G	LP	PM1, PM2, PM5, PP2, PP3, PP5	1 ^f^
*SOD1*	NM_000454	1	c.34G > T	p.D12Y	LP	PM1, PM2, PP2, PP3	3 ^f^
*SOD1*	NM_000454	3	c.203T > C	p.L68P	LP	PM1, PM2, PM5, PP2, PP5	1
*SOD1*	NM_000454	3	c.217G > A	p.G73S	P	PS1, PM1, PM2, PP2, PP3, PP5 (strong)	1
*SOD1*	NM_000454	4	c.255G > C	p.L85F	P	PS1, PM1 (strong), PM2, PM5, PP2, PP3	1 ^f^
*SOD1*	NM_000454	4	c.256G > A	p.G86S	LP	PM1, PM2, PM5, PP2, PP3	1
*SOD1*	NM_000454	4	c.272A > C	p.D91A	LP	PM1, PM2, PM5, PP2, PP5	2 ^#^
*SOD1*	NM_000454	4	c.281G > A	p.G94D	LP	PM1, PM2, PM5, PP2, PP3, PP5	1
*SOD1*	NM_000454	4	c.340A > T	p.I114F	LP	PM1, PM2, PM5, PP2, PP3, PP5	1 ^f^
*SOD1*	NM_000454	5	c.435G > C	p.L145F	P	PS1, PM1 (strong), PM2, PM5, PP2, PP3, PP5 (strong)	2 ^f^
*SOD1*	NM_000454	5	c.449T > C	p.I150T	LP	PM1, PM2, PP2, PP3, PP5	1 ^f^
*NEK1*	NM_001199397	29	c.2785_2786delGA	p.E929Nfs*12	P	PVS1 (very strong), PM2, PP3	1
*VCP*	NM_007126	5	c.463C > T	p.R155C	P	PM1 (strong), PM2, PM5, PP2, PP3, PP5 (very strong)	1

^#^ One patient was homozygous, the other heterozygous. ^f^ Familiarity for ALS present in 1 case. The evidence level considered is indicated in brackets, when different from what was expected for the criterion.

## Supplementary Materials

The following files are available online at https://www.mdpi.com/2073-4425/11/10/1123/s1. Table S1: List of coding variants with MAF < 1% identified in the 498 patients studied with gene panel sequencing. Table S2: List of the genes accounted for being somehow related to ALS pathogenesis, divided into four categories (i.e., Mendelian, susceptibility, modifier, and functionally involved).
