# Supplementary material for: High-Throughput Genetic Testing in ALS: The Challenging Path of Variant Classification Considering the ACMG Guidelines
Source: Genes (Basel). 2020 Sep 24;11(10):1123. doi: 10.3390/genes11101123 (PMC7600768; doi:10.3390/genes11101123)
Supplement: Supplementary file 1 [file genes-11-01123-s001.zip › Supplementary Table II.docx]

**Supplementary Table 2**

| **Gene** | **Chr** | **OMIM** | **Mendelian** | **Susceptibility** | **Modifier** | **Functional involvement** | **obs_mis** | **exp_mis** | **oe_mis upper** | **obs_lof** | **exp_lof** | **oe_lof**  **upper** | **pLI** | **OMIM** |
| --- | --- | --- | --- | --- | --- | --- | --- | --- | --- | --- | --- | --- | --- | --- |
| *SOD1* | 21 | 147450 | AD,AR [1] |  |  |  | 65 | 87 | 0.918 | 2 | 6.433 | 0.978 | 0.1773 |  |
| *ALS2* | 2 | 606352 | AR [2] |  |  |  | 713 | 870.46 | 0.871 | 36 | 92.2 | 0.516 | 0.0001 | Infantile onset ascending spastic paralysis |
| Locus 18q21 | 18 | 606640 | AD [3] |  |  |  |  |  |  |  |  |  |  |  |
| *SETX* | 9 | 608465 | AD [4] |  |  |  | 1392 | 1380.3 | 1.054 | 21 | 102.3 | 0.296 | 0.9548 | Spinocerebellar ataxia |
| *SPG11* | 15 | 610844 | AR [5,6] |  |  |  | 1360 | 1223 | 1.163 | 81 | 120 | 0.812 | 0.0001 | Charcot-Marie-Tooth disease axonal type 2X, spastic paraplegia 11 (AR), juvenile amyotrophic lateral sclerosis |
| *FUS* | 16 | 137070 | AD,(AR) [7,8] |  |  |  | 217 | 329.68 | 0.737 | 4 | 38.63 | 0.237 | 0.9993 | Hereditary essential tremor |
| Locus 20p13 | 20 | 608031 | AD [9] |  |  |  |  |  |  |  |  |  |  |  |
| *VAPB* | 20 | 605704 | AD [10] |  |  |  | 38 | 56.479 | 0.882 | 1 | 4.134 | 1.135 | 0.314 | Spinal muscular atrophy, late-onset |
| *ANG* | 14 | 105850 | AD, confl. [11] | Confl. [12,13] | Confl. [14] |  | 83 | 85.017 | 1.172 | 0 | 0.067 | 1.893 | 0.2873 |  |
| *TARDBP* | 1 | 605078 | AD [15,16] |  |  |  | 74 | 233.52 | 0.385 | 1 | 17.23 | 0.275 | 0.9854 | FTD |
| *FIG4* | 6 | 609390 | AD [17] |  |  |  | 381 | 498.9 | 0.831 | 52 | 52.81 | 1.24 | 0.0001 | Charcot-Marie-Tooth disease, type 4J(AR); Yunis-Varon syndrome (AR) |
| *OPTN* | 10 | 602432 | AD,AR [17] |  |  |  | 267 | 297.16 | 0.994 | 28 | 30.55 | 1.256 | 0.0001 | Open angle glaucoma |
| *VCP* | 9 | 601023 | AD [18] |  |  |  | 126 | 448.03 | 0.326 | 1 | 37.48 | 0.127 | 0.9999 | FTD, inclusion body myopathy, Paget’s disease, Charcot-Marie-Tooth disease, type 2Y Charcot-Marie-Tooth disease, type 2Y |
| *UBQLN2* | X | 300264 | XL [19] |  |  |  | 172 | 236.93 | 0.824 | 1 | 10.6 | 0.448 | 0.8496 |  |
| *SIGMAR1* | 9 | 601978 | AR [20] |  |  |  | 85 | 132.3 | 0.769 | 3 | 10.54 | 0.736 | 0.1678 | Juvenile amyotrophic lateral sclerosis, distal hereditary motor neuropathies |
| *CHMP2B* | 3 | 609512 | AD [21] |  |  |  | 103 | 113.15 | 1.072 | 10 | 13.95 | 1.216 | 0.0001 | FTD |
| *PFN1* | 17 | 176610 | AD [22] |  |  |  | 35 | 85.944 | 0.54 | 0 | 4.356 | 0.686 | 0.7325 |  |
| *ERBB4* | 2 | 600543 | AD [23] |  |  |  | 299 | 408.62 | 0.805 | 4 | 42.39 | 0.216 | 0.9998 |  |
| *HNRNPA1* | 12 | 164017 | AD [24] |  |  |  | 63 | 168.55 | 0.461 | 2 | 17.32 | 0.364 | 0.9307 | FTD, inclusion body myopathy, Paget’s disease |
| *HNRNPA2B1* | 7 | 600124 | AD [24] |  |  |  | 75 | 192.07 | 0.473 | 1 | 21.79 | 0.218 | 0.9973 | FTD, inclusion body myopathy, Paget’s disease |
| *MATR3* | 5 | 164015 | AD [25] |  |  |  | 289 | 452.38 | 0.704 | 0 | 37.52 | 0.079 | 1 | Distal myopathy with vocal cord and pharyngeal weakness |
| *TUBA4A* | 2 | 191110 | AD [26] |  |  |  | 120 | 273.78 | 0.51 | 4 | 14.76 | 0.62 | 0.1598 |  |
| *ANXA11* | 10 | 602572 | AD [27] |  |  |  | 311 | 298.91 | 1.143 | 19 | 28.45 | 0.98 | 0.0001 |  |
| *NEK1* | 4 | 604588 | AD [28,29] |  |  |  | 534 | 605.27 | 0.948 | 50 | 73.26 | 0.864 | 0.0001 | Short-rib thoracic dysplasia 6 with or without polydactyly (AR) |
| *KIF5A* | 12 | 602821 | AD [30,31] |  |  |  | 320 | 538.73 | 0.652 | 8 | 57.25 | 0.252 | 0.9995 | Spastic paraplegia 10 (AD), Neonatal intractable myoclonus, |
| *C9orf72* | 9 | 614260 | AD [32,33] |  |  |  | 105 | 115.97 | 1.065 | 8 | 9.763 | 1.468 | 0.0001 | FTD |
| *CHCHD10* | 22 | 615903 | AD, confl. [34,35] |  |  |  | 51 | 68.55 | 0.939 | 7 | 5.296 | 1.903 | 0.0001 | FTD, Spinal muscular atrophy Jokela type, isolated mitochondrial myopathy |
| *SQSTM1* | 5 | 601530 | AD [36] |  |  |  | 273 | 222.1 | 1.359 | 6 | 15.9 | 0.745 | 0.0106 | FTD, inclusion body myopathy, Paget’s disease, Neurodegeneration with ataxia, dystonia, and gaze palsy, childhood-onset |
| *TBK1* | 12 | 604834 | AD [37,38] |  |  |  | 267 | 371.96 | 0.794 | 11 | 43.34 | 0.42 | 0.0751 |  |
| *CCNF* | 16 | 600227 | AD [39] |  |  |  | 424 | 469.17 | 0.979 | 10 | 40.49 | 0.419 | 0.1267 |  |
| *ATP13A2* | 1 | 610513 | AR, n.r. [40] |  |  |  | 624 | 730.53 | 0.913 | 24 | 57.83 | 0.584 | 0.0001 | Kufor-Rakeb syndrome, spastic paraplegia 78 (AR) |
| *ATP7A* | X | 300011 | AD, n.r. [41] |  |  |  | 448 | 548.88 | 0.883 | 4 | 42.44 | 0.216 | 0.9998 | Menkes disease, occipital horn syndrome, spinal muscular atrophy (X-linked 3) |
| *C21orf2* | 21 | 603191 | AD, n.r. [42] |  |  |  | 169 | 159.28 | 1.205 | 8 | 10.67 | 1.35 | 0.0001 | Retinal dystrophy with macular staphyloma, axial spondylometaphyseal dysplasia |
| *CACNA1A* | 19 | 601011 | AD, confl. [43,44] |  |  |  | 844 | 1467.6 | 0.609 | 9 | 117.4 | 0.134 | 1 | Early infantile epileptic encephalopathy, episodic ataxia, migraine familial hemiplegic, spinocerebellar ataxia 6 |
| *CYLD* | 16 | 605018 | AD, n.r. [45] |  |  |  | 277 | 500.13 | 0.612 | 4 | 44.8 | 0.204 | 0.9999 | Familial cylindromatosis, Brooke-Spiegler syndrome, trichoepithelioma |
| *DAO* | 12 | 124050 | AD [46] |  |  |  | 196 | 200.29 | 1.102 | 29 | 23.14 | 1.697 | 0.0001 | Schizophrenia |
| *DCTN1* | 2 | 601143 | AD [47] |  |  |  | 642 | 708.85 | 0.967 | 17 | 70.13 | 0.364 | 0.0842 | Perry syndrome (AD),Neuronopathy, distal hereditary motor, type VIIB (AD) |
| *DNAJC7* | 17 | 601964 | AD, n.r. [48] |  |  |  | 130 | 242.07 | 0.621 | 3 | 27.67 | 0.28 | 0.992 |  |
| *ERLIN1* | 10 | 611604 | AR, n.r. [49] |  |  |  | 76 | 144.88 | 0.635 | 3 | 16.4 | 0.473 | 0.6465 | Spastic paraplegia 62 AR |
| *ERLIN2* | 8 | 611605 | AR,AD [50] |  |  |  | 108 | 178.64 | 0.709 | 8 | 17.32 | 0.833 | 0.0007 | Spastic paraplegia 18 (AR) |
| *EWSR1* | 22 | 133450 | n.r. [51] |  |  |  | 238 | 369.14 | 0.718 | 5 | 37.79 | 0.278 | 0.995 | Ewing sarcoma |
| *GARS* | 7 | 600287 | AD [52] |  |  | [53] | 334 | 401.58 | 0.911 | 9 | 39.05 | 0.402 | 0.3065 | Distal hereditary motor neuronopathy |
| *GLE1* | 9 | 603371 | AD [54] |  |  |  | 335 | 379.15 | 0.967 | 23 | 43.33 | 0.752 | 0.0001 | Lethal congenital contracture syndrome 1 (AR); Lethal arthrogryposis with anterior horn cell disease (AR) |
| *GLT8D1* | 3 | 618355 | AD, n.r. [55] |  |  |  | 179 | 203.95 | 0.993 | 15 | 20.36 | 1.134 | 0.0001 |  |
| *GRN* | 17 | 138945 | AD [56] |  |  |  | 341 | 355.59 | 1.049 | 8 | 29.9 | 0.483 | 0.0696 | Frontotemporal dementia, Neuronal ceroid lipofuscinosis, primary progressive aphasia |
| *MAPT* | 17 | 157140 | AD [57] |  |  |  | 372 | 464.17 | 0.873 | 9 | 26.33 | 0.596 | 0.0038 | FTD, Pick disease, Progressive supranuclear palsy |
| *NEFH* | 22 | 162230 | Confl.[58,59] |  |  |  | 470 | 503.02 | 1.008 | 22 | 29.53 | 1.064 | 0.0001 | Charcot-Marie-Tooth disease, axonal type 2CC |
| *PRPH* | 12 | 170710 | AD [60] |  |  |  | 245 | 270.73 | 1.006 | 21 | 21.86 | 1.383 | 0.0001 |  |
| *RAPGEF2* | 4 | 609530 | AD [61] |  |  |  | 532 | 824.32 | 0.693 | 8 | 73.13 | 0.197 | 1 |  |
| *SPAST* | 2 | 604277 | AD[62] |  |  |  | 279 | 343.91 | 0.896 | 3 | 34.56 | 0.224 | 0.9993 | Spastic paraplegia 4 (AD) |
| *SPG7* | 16 | 602783 | AD [63,64] |  |  |  | 528 | 474.55 | 1.196 | 48 | 36.95 | 1.651 | 0.0001 | Spastic paraplegia 7 (AR) |
| *SS18L1* | 20 | 606472 | AD [65] |  |  |  | 184 | 254.29 | 0.818 | 4 | 29.93 | 0.306 | 0.9822 |  |
| *TAF15* | 17 | 601574 | AD, n.r. [66] |  |  |  | 284 | 348.03 | 0.9 | 11 | 45.61 | 0.399 | 0.1593 | Chondrosarcoma, extraskeletal myxoid |
| *TIA1* | 2 | 603518 | AD, confl. [67,68] |  |  |  | 128 | 213.62 | 0.694 | 7 | 29.7 | 0.443 | 0.2656 | Welander distal myopathy |
| *VRK1* | 14 | 602168 | AR, [69] |  |  |  | 167 | 215.17 | 0.882 | 9 | 24.78 | 0.634 | 0.0022 | Pontocerebellar hypoplasia type 1A (AR) |
| *ARHGEF28* | 5 | 612790 | AD, confl. [70,71] |  |  |  | 806 | 860.89 | 0.992 | 47 | 82.83 | 0.724 | 0.0001 |  |
| *ARPP21* | 3 | 605488 | n.r. [72] |  |  |  | 431 | 448.3 | 1.041 | 15 | 48.27 | 0.478 | 0.0008 |  |
| *C19orf12* | 19 | 614297 | Mimicking [73–75] |  |  |  | 79 | 87.933 | 1.083 | 1 | 3.352 | 1.355 | 0.2457 | Neurodegeneration with brain iron accumulation (AR); ?Spastic paraplegia 43 (AR) |
| *CACNA1H* | 16 | 607904 | AD, n.r. [76] |  |  |  | 1717 | 1462.9 | 1.221 | 32 | 85.27 | 0.504 | 0.0001 | {Epilepsy, childhood absence, susceptibility to, 6}, Hyperaldosteronism, familial, type IV, (AD) |
| *HEXA* | 15 | 606869 | Mimicking [77,78] |  |  |  | 297 | 277.81 | 1.177 | 22 | 32.3 | 0.973 | 0.0001 | GM2-gangliosidosis (AR); Tay-Sachs disease (AR) |
| *LUM* | 12 | 600616 | n.r. [79] |  |  |  | 113 | 174.39 | 0.758 | 3 | 8.272 | 0.937 | 0.0769 |  |
| *MYH15* | 3 | 609929 | n.r. [80] |  |  |  | 998 | 989.26 | 1.063 | 96 | 107.2 | 1.061 | 0.0001 |  |
| *NAIP* | 5 | 600355 | AR, n.r. [81] |  |  | [82] | 107 | 119.6 | 1.05 | 7 | 10.04 | 1.306 | 0.0002 | Charcot-Marie-Tooth disease, recessive intermediate C (AR) Spinal muscular atrophy, distal (AR) |
| *PLEKHG5* | 1 | 611101 | n.r. [83] |  |  |  | 512 | 610.98 | 0.902 | 17 | 43.98 | 0.58 | 0.0001 | Boucher-Neuhauser syndrome, 215470 (3), Autosomal recessive; ?Laurence-Moon syndrome, 245800 (3), Autosomal recessive; Oliver-McFarlane syndrome, 275400 (3), Autosomal recessive; Spastic paraplegia 39, autosomal recessive, 612020 (3), Autosomal recessive |
| *PNPLA6* | 19 | 603197 | AR, n.r. [84] |  |  |  | 505 | 866.88 | 0.627 | 31 | 67.84 | 0.617 | 0.0001 | {Amyotrophic lateral sclerosis, susceptibility to}, 105400 (3), Autosomal recessive, Autosomal dominant |
| *SYNE1* | 6 | 608441 | n.r. [83] |  |  |  | 4465 | 4409.5 | 1.038 | 181 | 487.2 | 0.42 | 0.0001 | Emery-Dreifuss muscular dystrophy 4, autosomal dominant, 612998 (3), Autosomal dominant; Spinocerebellar ataxia, autosomal recessive 8, 610743 (3), Autosomal recessive |
| *TFG* | 3 | 602498 | n.r. [85] |  |  |  | 166 | 221.78 | 0.851 | 6 | 23.17 | 0.511 | 0.1406 | Hereditary motor and sensory neuropathy, Okinawa type (AD) ?Spastic paraplegia 57 (AR) |
| *TRPM7* | 15 | 605692 | Confl. [86,87] |  |  |  | 756 | 949.65 | 0.845 | 44 | 101.5 | 0.558 | 0.0001 |  |
| *UBQLN1* | 9 | 605046 | Confl. [88,89] |  |  |  | 180 | 299.52 | 0.68 | 2 | 26.1 | 0.241 | 0.9972 |  |
| *ADRB3* | 8 | 109691 |  | n.r. [90] |  |  | 214 | 231.37 | 1.036 | 10 | 9.782 | 1.683 | 0.0001 |  |
| *ALAD* | 9 | 125270 |  | n.r. [91] |  |  | 150 | 196.9 | 0.872 | 6 | 18.19 | 0.651 | 0.0247 | Porphyria, acute hepatic (AR) |
| *APEX1* | 14 | 107748 |  | Confl. [92,93] |  |  | 172 | 176.51 | 1.106 | 12 | 15.27 | 1.273 | 0.0001 |  |
| *APOE* | 19 | 107741 |  | Confl. [94,95] |  |  | 188 | 218.82 | 0.97 | 6 | 11.46 | 1.034 | 0.0019 | Alzheimer disease-2 (AD), Hyperlipoproteinemia, type III, Lipoprotein glomerulopathy, Sea-blue histiocyte disease (AR) |
| *ATXN1* |  |  |  | [96,97] |  |  |  |  |  |  |  |  | 0 |  |
| *ATXN2* | 12 | 601517 |  | [98,99] | [100] |  | 466 | 632.04 | 0.796 | 11 | 54.75 | 0.333 | 0.8528 | Spinocerebellar ataxia (AD) |
| *C8orf46* | 8 | . |  | n.r. [101] |  |  | 91 | 121.87 | 0.889 | 3 | 9.756 | 0.795 | 0.1299 |  |
| *CHGB* | 20 | 118920 |  | Confl. [102,103] |  |  | 358 | 362.5 | 1.078 | 16 | 31.32 | 0.776 | 0.0001 |  |
| *CHRNA3* | 15 | 118503 |  | n.r. [104] |  |  | 247 | 281.71 | 0.974 | 13 | 18 | 1.148 | 0.0001 |  |
| *CHRNA4* | 20 | 118504 |  | [105] |  |  | 377 | 396.13 | 1.036 | 10 | 20.64 | 0.822 | 0.0001 | Epilepsy, nocturnal frontal lobe (AD) |
| *CHRNB4* | 15 | 118509 |  | n.r. [104] |  |  | 257 | 316.86 | 0.899 | 5 | 18.81 | 0.559 | 0.1427 |  |
| *CNTN4* | 3 | 607280 |  | n.r. [106] |  |  | 416 | 444.67 | 1.015 | 15 | 44.67 | 0.517 | 0.0002 |  |
| *CX3CR1* | 3 | 601470 |  | n.r. [107] |  |  | 162 | 205.59 | 0.898 | 3 | 7.672 | 1.01 | 0.0613 |  |
| *CYP2D6* | 22 | 124030 |  | n.r. [108] |  |  | 350 | 268.16 | 1.426 | 27 | 16.2 | 1.951 | 0.0001 |  |
| *DISC1* | 1 | 605210 |  | n.r. [109] |  |  | 219 | 217.55 | 1.126 | 4 | 8.918 | 1.026 | 0.0195 |  |
| *DPP6* | 7 | 126141 |  | Confl. [110,111] |  |  | 312 | 451.02 | 0.76 | 12 | 48.76 | 0.399 | 0.1058 | Mental retardation (AD) |
| *DPYSL3* | 5 | 601168 |  | n.r. [112] |  |  | 271 | 412.64 | 0.726 | 2 | 28.52 | 0.221 | 0.9988 |  |
| *DYNC1H1* | 14 | 600112 |  | Confl. [113,114] |  |  | 1022 | 2593.9 | 0.415 | 11 | 228.1 | 0.08 | 1 | Charcot-Marie-Tooth disease, axonal, type 20 (AD), Mental retardation (AD), Spinal muscular atrophy, lower extremity-predominant 1 (AD) |
| *ELP3* | 8 | 612722 |  | n.r. [115] |  |  | 228 | 320.18 | 0.795 | 26 | 35.21 | 1.025 | 0.0001 |  |
| *FGGY* | 1 | 611370 |  | Confl. [116,117] |  |  | 320 | 311.67 | 1.126 | 29 | 31.07 | 1.273 | 0.0001 |  |
| *HFE* | 6 | 613609 |  | Confl. [118,119] |  |  | 160 | 181.39 | 1.006 | 14 | 18.48 | 1.185 | 0.0001 | Hemochromatosis (AR) |
| *ITPR2* | 12 | 600144 |  | Confl. [120,121] |  |  | 50 | 96.585 | 0.655 | 3 | 10.03 | 0.773 | 0.142 |  |
| *LIPC* | 3 | 607365 |  | n.r. [90] |  |  | 210 | 242.43 | 0.971 | 18 | 22.08 | 0.001 | 0 |  |
| *MOBP* | 3 | 600948 |  | n.r. [42] |  |  | 72 | 132.01 | 0.664 | 1 | 7.793 | 0.609 | 0.6617 |  |
| *MMP3* | 11 | 185250 |  | n.r. [90] |  |  | 285 | 256 | 1.228 | 22 | 22.17 | 1.416 | 0.0001 |  |
| *NIPA1* | 15 | 608145 |  | [122] |  |  | 110 | 184.14 | 0.7 | 5 | 10.11 | 1.04 | 0.0059 | Spastic paraplegia 6 (AD) |
| *PON1*  *PON2*  *PON3* | 7 | 168820  602447  602720 |  | [123] |  |  | 151 | 178.22 | 0.97 | 17 | 19.77 | 1.29 | 0.0001 |  |
| *PSEN1* | 14 | 104311 |  | n.r. [124] |  |  | 125 | 221.62 | 0.654 | 3 | 20.67 | 0.375 | 0.9006 | Acne inversa, familial, 3 (AD), Alzheimer disease, type 3 (AD) |
| *PVR* | 19 | 173850 |  | n.r. [125] |  |  | 180 | 220.98 | 0.922 | 14 | 16.24 | 1.347 | 0.0001 |  |
| *RAD9B* | 12 | 608368 |  | [101] |  |  | 187 | 199.77 | 1.057 | 20 | 21.24 | 1.368 | 0.0001 |  |
| *SCFD1* | 14 | 618207 |  | n.r. [42] |  |  | 180 | 279.14 | 0.73 | 5 | 40.2 | 0.262 | 0.9979 |  |
| *SLC1A2* | 11 | 600300 |  | Confl. |  |  | 217 | 334.6 | 0.726 | 4 | 21.59 | 0.424 | 0.7103 |  |
| *SMN1* | 5 | 600354 |  | [126] |  |  | 7 | 7.4146 | 1.689 | 2 | 1.245 | 1.929 | 0.0158 |  |
| *SMN2* | 5 | 601627 |  | Confl. [127,128] |  |  | 0 | 0.8256 | 1.768 | 1 | 0.179 | 1.942 | 0.0582 |  |
| *TREM2* | 6 | 605086 |  | [129] |  |  | 128 | 125.85 | 1.178 | 8 | 7.672 | 1.757 | 0.0001 | Polycystic lipomembranous osteodysplasia with sclerosing leukoencephalopathy 2 (AD) |
| *UNC13A* | 19 | 609894 |  | [130] |  |  | 513 | 1017.4 | 0.542 | 8 | 96.94 | 0.149 | 1 |  |
| *VDR* | 12 | 601769 |  | n.r. [91] |  |  | 207 | 259.16 | 0.896 | 8 | 17.54 | 0.823 | 0.0008 | Rickets, vitamin D-resistant, type IIA (AR) |
| *VEGFA* | 6 | 192240 |  | [131] |  |  | 67 | 99.045 | 0.829 | 1 | 11.48 | 0.413 | 0.8871 |  |
| *ZNF512B* | 20 | 617886 |  | [132] | [133] |  | 450 | 572.99 | 0.849 | 4 | 44.34 | 0.206 | 0.9999 |  |
| *CAMTA1* | 1 | 611501 |  |  | n.r. [134] |  | 725 | 1017.9 | 0.757 | 7 | 76.63 | 0.172 | 1 | Cerebellar ataxia, nonprogressive, with mental retardation (AD) |
| *EPHA3* | 3 | 179611 |  |  | n.r. [135] |  | 503 | 545.87 | 0.992 | 18 | 50.61 | 0.527 | 0.0001 |  |
| *KIFAP3* | 1 | 601836 |  |  | Confl. [136,137] |  | 295 | 402.86 | 0.806 | 17 | 46.95 | 0.543 | 0.0001 |  |
| *LIF* | 22 | 159540 |  |  | n.r. [138] |  | 109 | 121.32 | 1.053 | 1 | 6.304 | 0.752 | 0.5242 |  |
| *ADCYAP1* | 18 | 102980 |  |  |  | [139] | 112 | 99.832 | 1.313 | 1 | 6.304 | 0.752 | 0.5242 |  |
| *AGT* | 1 | 618355 |  |  |  | [140] | 275 | 265.86 | 1.143 | 12 | 13.43 | 1.445 | 0.0001 |  |
| *AR* | X | 313700 |  |  |  | Confl. [141,142] | 286 | 350.67 | 0.899 | 3 | 26.66 | 0.291 | 0.9883 | Androgen insensitivity (XL), Spinal and bulbar muscular atrophy of Kennedy (XL) |
| *BCL11B* | 14 | 606558 |  |  |  | [143] | 204 | 509.49 | 0.45 | 1 | 19.79 | 0.24 | 0.9943 |  |
| *CCS* | 11 | 603864 |  |  |  | [144] | 150 | 161.13 | 1.066 | 12 | 12.59 | 1.534 | 0.0001 |  |
| *CNTF* | 11 | 118945 |  |  |  | [145] | 139 | 118.96 | 1.345 | 7 | 7.025 | 1.743 | 0.0001 |  |
| *CRYM* | 16 | 123740 |  |  |  | [146] | 138 | 174.61 | 0.91 | 5 | 13.91 | 0.756 | 0.0256 | Deafness, autosomal dominant 40 |
| *CST3* | 20 | 604312 |  |  |  | [147] | 81 | 69.363 | 1.405 | 3 | 3.689 | 1.766 | 0.0098 | Cerebral amyloid angiopathy (AD) |
| *EPHA4* | 2 | 602188 |  |  |  | [148] | 377 | 571.02 | 0.719 | 4 | 52.18 | 0.175 | 1 |  |
| *HSPB1* | 7 | 602195 |  |  |  | [149] | 136 | 120.13 | 1.305 | 9 | 7.171 | 1.872 | 0.0001 | Charcot-Marie-Tooth disease, axonal, type 2F (AD), Neuropathy, distal hereditary motor, type IIB (AD) |
| *KDR* | 4 | 191306 |  |  |  | [150] | 659 | 737.5 | 0.953 | 11 | 73.04 | 0.249 | 0.9998 | Hemangioma, capillary infantile, somatic |
| *LIPC* | 15 | 151670 |  |  |  | [90] | 335 | 290.91 | 1.261 | 14 | 23.54 | 0.93 | 0.0001 | Hepatic lipase deficiency (AR) |
| *LMNB1* | 5 | 150340 |  |  |  | [151] | 229 | 309.52 | 0.825 | 6 | 28.6 | 0.414 | 0.5548 | Leukodystrophy, adult-onset (AD) |
| *LOX* | 5 | 153455 |  |  |  | [152] | 205 | 233.02 | 0.988 | 2 | 22.09 | 0.285 | 0.9876 |  |
| *MAOB* | X | 309860 |  |  |  | [153] | 118 | 185 | 0.743 | 0 | 20.11 | 0.148 | 0.999 |  |
| *MAP4K4* | 2 | 604666 |  |  |  | [154,155] | 289 | 558.56 | 0.57 | 6 | 64.25 | 0.184 | 1 |  |
| *NDRG2* | 14 | 605272 |  |  |  | [156] | 174 | 194.27 | 1.016 | 10 | 21.46 | 0.79 | 0.0002 |  |
| *OGG1* | 3 | 601982 |  |  |  | [157] | 267 | 238.9 | 1.237 | 18 | 20.84 | 1.281 | 0.0001 | Renal cell carcinoma, clear cell, somatic |
| *PARK7* | 1 | 602533 |  |  |  | [158] | 104 | 108.83 | 1.125 | 1 | 8.966 | 0.529 | 0.7533 | Parkinson disease 7, autosomal recessive early-onset |
| *PCP4* | 21 | 601629 |  |  |  | [159] | 24 | 34.069 | 0.991 | 4 | 4.466 | 1.773 | 0.0022 |  |
| *PPARGC1A* | 4 | 604517 |  |  |  | [160] | 385 | 444.43 | 0.943 | 5 | 39.4 | 0.267 | 0.9973 |  |
| *RAMP3* | 7 | 605155 |  |  |  | [101] | 74 | 88.811 | 1.011 | 3 | 4.656 | 1.574 | 0.0163 |  |
| *RNF19A* | 8 | 607119 |  |  |  | [161] | 322 | 466.69 | 0.757 | 10 | 35.88 | 0.473 | 0.0253 |  |
| *SARM1* | 17 | 607732 |  |  |  | [162] | 269 | 372.35 | 0.799 | 18 | 23.36 | 1.143 | 0.0001 |  |
| *SCN7A* | 2 | 182392 |  |  |  | [163] | 761 | 793.27 | 1.019 | 37 | 62.1 | 0.784 | 0.0001 |  |
| *SELL* | 1 | 153240 |  |  |  | [164] | 179 | 191.45 | 1.058 | 13 | 20.47 | 1.01 | 0.0001 |  |
| *SFPQ* | 1 | 605199 |  |  |  | [165] | 187 | 341.96 | 0.617 | 1 | 31.31 | 0.152 | 0.9999 |  |
| *SIRT3* | 11 | 604481 |  |  |  | [166] | 226 | 227.11 | 1.111 | 8 | 13.81 | 1.045 | 0.0002 |  |
| *SNCG* | 10 | 602998 |  |  |  | [167] | 74 | 81.525 | 1.101 | 7 | 8.966 | 1.455 | 0.0001 |  |
| *SOD2* | 6 | 147460 |  |  |  | [168] | 84 | 98.239 | 1.025 | 3 | 8.335 | 0.93 | 0.0787 |  |
| *SOX5* | 12 | 604975 |  |  |  | [169] | 242 | 420.64 | 0.64 | 2 | 39.78 | 0.158 | 0.9999 | Lamb-Shaffer syndrome (AD) |
| *VPS54* | 2 | 614633 |  |  |  | [170,171] | 425 | 483.84 | 0.952 | 7 | 51.82 | 0.254 | 0.9992 |  |
| *VRK1* | 14 | 602168 |  |  |  | [69] | 167 | 215.17 | 0.882 | 9 | 24.78 | 0.634 | 0.0022 | Pontocerebellar hypoplasia type 1A (AR) |

Genes are divided into four categories: divided into four categories: Mendelian, if variants suspected to be causative for a Mendelian form of ALS have been reported; Susceptibility: if variants have been detected for which a statistically significant association with an increased risk of developing ALS has been found; Modifier: if they have been claimed to modify some aspects of the ALS phenotype (e.g.: age of onset, severity, survival); Functionally involved: if no genetic variant has been identified yet to be someway associated with ALS, by functional studies support the involvement of the gene product in the ALS pathogenesis.

Confl.: different studies reached conflicting evidences about the suggested involvement of the gene in ALS.

N.r.: not replicated. It means the after an initial study suggesting a certain role for the gene in ALS, further confirmatory evidences are still awaited.

Seven gnomAD gene constraint metrics are reported [172]:

1. “obs_mis: Number of observed missense variants in the canonical transcript of the gene”
2. “exp_mis: Number of expected missense variants in the canonical transcript of the gene”
3. “oe_mis upper: Upper bound of 90% confidence interval for observed/expected ratio for missense variants. Lower values indicate more constrained genes”
4. "obs_lof: Number of observed predicted loss-of-function (pLoF) variants in transcript in the canonical transcript of the gene”
5. "exp_lof: Number of expected predicted loss-of-function (pLoF) variants in transcript in the canonical transcript of the gene”
6. “oe_lof_upper: LOEUF: upper bound of 90% confidence interval for o/e ratio for pLoF (predicted loss-of-function) variants. Lower values indicate more constrained genes.”
7. “pLI: Probability of loss-of-function intolerance; probability that transcript falls into distribution of haploinsufficient genes.” Values can go from 0 (the gene is not loss-of-function intolerant) to 1 (the maximum likelihood that the gene is loss-of-function intolerant).

**References**

1. Rosen, D.R.; Siddique, T.; Patterson, D.; Figlewicz, D.A.; Sapp, P.; Hentati, A.; Donaldson, D.; Goto, J.; O’Regan, J.P.; Deng, H.-X.; et al. Mutations in Cu/Zn superoxide dismutase gene are associated with familial amyotrophic lateral sclerosis. *Nature* **1993**, *362*, 59–62, doi:10.1038/362059a0.

2. Hadano, S.; Yanagisawa, Y.; Skaug, J.; Fichter, K.; Nasir, J.; Martindale, D.; Koop, B.F.; Scherer, S.W.; Nicholson, D.W.; Rouleau, G.A.; et al. Cloning and Characterization of Three Novel Genes, ALS2CR1, ALS2CR2, and ALS2CR3, in the Juvenile Amyotrophic Lateral Sclerosis (ALS2) Critical Region at Chromosome 2q33–q34: Candidate Genes for ALS2. *Genomics* **2001**, *71*, 200–213, doi:10.1006/geno.2000.6392.

3. Hand, C.K.; Khoris, J.; Salachas, F.; Gros-Louis, F.; Lopes, A.A.S.; Mayeux-Portas, V.; Brown, R.H.; Meininger, V.; Camu, W.; Rouleau, G.A. A Novel Locus for Familial Amyotrophic Lateral Sclerosis, on Chromosome 18q. *Am. J. Hum. Genet.* **2002**, *70*, 251–256, doi:10.1086/337945.

4. Chen, Y.-Z.; Bennett, C.L.; Huynh, H.M.; Blair, I.P.; Puls, I.; Irobi, J.; Dierick, I.; Abel, A.; Kennerson, M.L.; Rabin, B.A.; et al. DNA/RNA Helicase Gene Mutations in a Form of Juvenile Amyotrophic Lateral Sclerosis (ALS4). *Am. J. Hum. Genet.* **2004**, *74*, 1128–1135, doi:10.1086/421054.

5. Orlacchio, A.; Babalini, C.; Borreca, A.; Patrono, C.; Massa, R.; Basaran, S.; Munhoz, R.P.; Rogaeva, E.A.; St George-Hyslop, P.H.; Bernardi, G.; et al. SPATACSIN mutations cause autosomal recessive juvenile amyotrophic lateral sclerosis. *Brain* **2010**, *133*, 591–598, doi:10.1093/brain/awp325.

6. Daoud, H.; Zhou, S.; Noreau, A.; Sabbagh, M.; Belzil, V.; Dionne-Laporte, A.; Tranchant, C.; Dion, P.; Rouleau, G.A. Exome sequencing reveals SPG11 mutations causing juvenile ALS. *Neurobiol. Aging* **2012**, *33*, 839.e5-839.e9, doi:10.1016/j.neurobiolaging.2011.11.012.

7. Vance, C.; Rogelj, B.; Hortobagyi, T.; De Vos, K.J.; Nishimura, A.L.; Sreedharan, J.; Hu, X.; Smith, B.; Ruddy, D.; Wright, P.; et al. Mutations in FUS, an RNA Processing Protein, Cause Familial Amyotrophic Lateral Sclerosis Type 6. *Science (80-. ).* **2009**, *323*, 1208–1211, doi:10.1126/science.1165942.

8. Kwiatkowski, T.J.; Bosco, D.A.; LeClerc, A.L.; Tamrazian, E.; Vanderburg, C.R.; Russ, C.; Davis, A.; Gilchrist, J.; Kasarskis, E.J.; Munsat, T.; et al. Mutations in the FUS/TLS Gene on Chromosome 16 Cause Familial Amyotrophic Lateral Sclerosis. *Science (80-. ).* **2009**, *323*, 1205–1208, doi:10.1126/science.1166066.

9. Sapp, P.C.; Hosler, B.A.; McKenna-Yasek, D.; Chin, W.; Gann, A.; Genise, H.; Gorenstein, J.; Huang, M.; Sailer, W.; Scheffler, M.; et al. Identification of Two Novel Loci for Dominantly Inherited Familial Amyotrophic Lateral Sclerosis. *Am. J. Hum. Genet.* **2003**, *73*, 397–403, doi:10.1086/377158.

10. Nishimura, A.L.; Mitne-Neto, M.; Silva, H.C.A.; Richieri-Costa, A.; Middleton, S.; Cascio, D.; Kok, F.; Oliveira, J.R.M.; Gillingwater, T.; Webb, J.; et al. A Mutation in the Vesicle-Trafficking Protein VAPB Causes Late-Onset Spinal Muscular Atrophy and Amyotrophic Lateral Sclerosis. *Am. J. Hum. Genet.* **2004**, *75*, 822–831, doi:10.1086/425287.

11. Greenway, M.J.; Andersen, P.M.; Russ, C.; Ennis, S.; Cashman, S.; Donaghy, C.; Patterson, V.; Swingler, R.; Kieran, D.; Prehn, J.; et al. ANG mutations segregate with familial and “sporadic” amyotrophic lateral sclerosis. *Nat. Genet.* **2006**, *38*, 411–413, doi:10.1038/ng1742.

12. Pan, L.; Deng, X.; Ding, D.; Leng, H.; Zhu, X.; Wang, Z. Association between the Angiogenin (ANG) K17I variant and amyotrophic lateral sclerosis risk in Caucasian: a meta-analysis. *Neurol. Sci.* **2015**, *36*, 2163–2168, doi:10.1007/s10072-015-2344-5.

13. Pan, L.; Deng, X.; Wang, Z.; Leng, H.; Zhu, X.; Ding, D. Lack of association between the Angiogenin (ANG) rs11701 polymorphism and amyotrophic lateral sclerosis risk: a meta-analysis. *Neurol. Sci.* **2016**, *37*, 655–662, doi:10.1007/s10072-015-2473-x.

14. Aluri, K.C.; Salisbury, J.P.; Prehn, J.H.M.; Agar, J.N. Loss of angiogenin function is related to earlier ALS onset and a paradoxical increase in ALS duration. *Sci. Rep.* **2020**, *10*, 3715, doi:10.1038/s41598-020-60431-6.

15. Kabashi, E.; Valdmanis, P.N.; Dion, P.; Spiegelman, D.; McConkey, B.J.; Velde, C. Vande; Bouchard, J.-P.; Lacomblez, L.; Pochigaeva, K.; Salachas, F.; et al. TARDBP mutations in individuals with sporadic and familial amyotrophic lateral sclerosis. *Nat. Genet.* **2008**, *40*, 572–574, doi:10.1038/ng.132.

16. Sreedharan, J.; Blair, I.P.; Tripathi, V.B.; Hu, X.; Vance, C.; Rogelj, B.; Ackerley, S.; Durnall, J.C.; Williams, K.L.; Buratti, E.; et al. TDP-43 Mutations in Familial and Sporadic Amyotrophic Lateral Sclerosis. *Science (80-. ).* **2008**, *319*, 1668–1672, doi:10.1126/science.1154584.

17. Maruyama, H.; Morino, H.; Ito, H.; Izumi, Y.; Kato, H.; Watanabe, Y.; Kinoshita, Y.; Kamada, M.; Nodera, H.; Suzuki, H.; et al. Mutations of optineurin in amyotrophic lateral sclerosis. *Nature* **2010**, *465*, 223–226, doi:10.1038/nature08971.

18. Johnson, J.O.; Mandrioli, J.; Benatar, M.; Abramzon, Y.; Van Deerlin, V.M.; Trojanowski, J.Q.; Gibbs, J.R.; Brunetti, M.; Gronka, S.; Wuu, J.; et al. Exome Sequencing Reveals VCP Mutations as a Cause of Familial ALS. *Neuron* **2010**, *68*, 857–864, doi:10.1016/j.neuron.2010.11.036.

19. Deng, H.-X.; Chen, W.; Hong, S.-T.; Boycott, K.M.; Gorrie, G.H.; Siddique, N.; Yang, Y.; Fecto, F.; Shi, Y.; Zhai, H.; et al. Mutations in UBQLN2 cause dominant X-linked juvenile and adult-onset ALS and ALS/dementia. *Nature* **2011**, *477*, 211–215, doi:10.1038/nature10353.

20. Al-Saif, A.; Al-Mohanna, F.; Bohlega, S. A mutation in sigma-1 receptor causes juvenile amyotrophic lateral sclerosis. *Ann. Neurol.* **2011**, *70*, 913–919, doi:10.1002/ana.22534.

21. Parkinson, N.; Ince, P.G.; Smith, M.O.; Highley, R.; Skibinski, G.; Andersen, P.M.; Morrison, K.E.; Pall, H.S.; Hardiman, O.; Collinge, J.; et al. ALS phenotypes with mutations in CHMP2B (charged multivesicular body protein 2B). *Neurology* **2006**, *67*, 1074–1077, doi:10.1212/01.wnl.0000231510.89311.8b.

22. Wu, C.-H.; Fallini, C.; Ticozzi, N.; Keagle, P.J.; Sapp, P.C.; Piotrowska, K.; Lowe, P.; Koppers, M.; McKenna-Yasek, D.; Baron, D.M.; et al. Mutations in the profilin 1 gene cause familial amyotrophic lateral sclerosis. *Nature* **2012**, *488*, 499–503, doi:10.1038/nature11280.

23. Takahashi, Y.; Fukuda, Y.; Yoshimura, J.; Toyoda, A.; Kurppa, K.; Moritoyo, H.; Belzil, V.V.; Dion, P.A.; Higasa, K.; Doi, K.; et al. ERBB4 Mutations that Disrupt the Neuregulin-ErbB4 Pathway Cause Amyotrophic Lateral Sclerosis Type 19. *Am. J. Hum. Genet.* **2013**, *93*, 900–905, doi:10.1016/j.ajhg.2013.09.008.

24. Kim, H.J.; Kim, N.C.; Wang, Y.-D.; Scarborough, E.A.; Moore, J.; Diaz, Z.; MacLea, K.S.; Freibaum, B.; Li, S.; Molliex, A.; et al. Mutations in prion-like domains in hnRNPA2B1 and hnRNPA1 cause multisystem proteinopathy and ALS. *Nature* **2013**, *495*, 467–473, doi:10.1038/nature11922.

25. Johnson, J.O.; Pioro, E.P.; Boehringer, A.; Chia, R.; Feit, H.; Renton, A.E.; Pliner, H.A.; Abramzon, Y.; Marangi, G.; Winborn, B.J.; et al. Mutations in the Matrin 3 gene cause familial amyotrophic lateral sclerosis. *Nat. Neurosci.* **2014**, *17*, 664–666, doi:10.1038/nn.3688.

26. Smith, B.N.; Ticozzi, N.; Fallini, C.; Gkazi, A.S.; Topp, S.; Kenna, K.P.; Scotter, E.L.; Kost, J.; Keagle, P.; Miller, J.W.; et al. Exome-wide Rare Variant Analysis Identifies TUBA4A Mutations Associated with Familial ALS. *Neuron* **2014**, *84*, 324–331, doi:10.1016/j.neuron.2014.09.027.

27. Smith, B.N.; Topp, S.D.; Fallini, C.; Shibata, H.; Chen, H.-J.; Troakes, C.; King, A.; Ticozzi, N.; Kenna, K.P.; Soragia-Gkazi, A.; et al. Mutations in the vesicular trafficking protein annexin A11 are associated with amyotrophic lateral sclerosis. *Sci. Transl. Med.* **2017**, *9*, eaad9157, doi:10.1126/scitranslmed.aad9157.

28. Brenner, D.; Müller, K.; Wieland, T.; Weydt, P.; Böhm, S.; Lulé, D.; Hübers, A.; Neuwirth, C.; Weber, M.; Borck, G.; et al. *NEK1* mutations in familial amyotrophic lateral sclerosis. *Brain* **2016**, *139*, e28–e28, doi:10.1093/brain/aww033.

29. Kenna, K.P.; van Doormaal, P.T.C.; Dekker, A.M.; Ticozzi, N.; Kenna, B.J.; Diekstra, F.P.; van Rheenen, W.; van Eijk, K.R.; Jones, A.R.; Keagle, P.; et al. NEK1 variants confer susceptibility to amyotrophic lateral sclerosis. *Nat. Genet.* **2016**, *48*, 1037–1042, doi:10.1038/ng.3626.

30. Nicolas, A.; Kenna, K.P.; Renton, A.E.; Ticozzi, N.; Faghri, F.; Chia, R.; Dominov, J.A.; Kenna, B.J.; Nalls, M.A.; Keagle, P.; et al. Genome-wide Analyses Identify KIF5A as a Novel ALS Gene. *Neuron* **2018**, *97*, 1268-1283.e6, doi:10.1016/j.neuron.2018.02.027.

31. Brenner, D.; Yilmaz, R.; Müller, K.; Grehl, T.; Petri, S.; Meyer, T.; Grosskreutz, J.; Weydt, P.; Ruf, W.; Neuwirth, C.; et al. Hot-spot KIF5A mutations cause familial ALS. *Brain* **2018**, *141*, 688–697, doi:10.1093/brain/awx370.

32. Renton, A.E.; Majounie, E.; Waite, A.; Simón-Sánchez, J.; Rollinson, S.; Gibbs, J.R.; Schymick, J.C.; Laaksovirta, H.; van Swieten, J.C.; Myllykangas, L.; et al. A Hexanucleotide Repeat Expansion in C9ORF72 Is the Cause of Chromosome 9p21-Linked ALS-FTD. *Neuron* **2011**, *72*, 257–268, doi:10.1016/j.neuron.2011.09.010.

33. DeJesus-Hernandez, M.; Mackenzie, I.R.; Boeve, B.F.; Boxer, A.L.; Baker, M.; Rutherford, N.J.; Nicholson, A.M.; Finch, N.A.; Flynn, H.; Adamson, J.; et al. Expanded GGGGCC Hexanucleotide Repeat in Noncoding Region of C9ORF72 Causes Chromosome 9p-Linked FTD and ALS. *Neuron* **2011**, *72*, 245–256, doi:10.1016/j.neuron.2011.09.011.

34. Bannwarth, S.; Ait-El-Mkadem, S.; Chaussenot, A.; Genin, E.C.; Lacas-Gervais, S.; Fragaki, K.; Berg-Alonso, L.; Kageyama, Y.; Serre, V.; Moore, D.G.; et al. A mitochondrial origin for frontotemporal dementia and amyotrophic lateral sclerosis through CHCHD10 involvement. *Brain* **2014**, *137*, 2329–2345, doi:10.1093/brain/awu138.

35. Project MinE ALS Sequencing Consortium *CHCHD10* variants in amyotrophic lateral sclerosis: Where is the evidence? *Ann. Neurol.* **2018**, *84*, 110–116, doi:10.1002/ana.25273.

36. Fecto, F. &lt;emph type="ital"&gt;SQSTM1&lt;/emph&gt; Mutations in Familial and Sporadic Amyotrophic Lateral Sclerosis. *Arch. Neurol.* **2011**, *68*, 1440, doi:10.1001/archneurol.2011.250.

37. Freischmidt, A.; Wieland, T.; Richter, B.; Ruf, W.; Schaeffer, V.; Müller, K.; Marroquin, N.; Nordin, F.; Hübers, A.; Weydt, P.; et al. Haploinsufficiency of TBK1 causes familial ALS and fronto-temporal dementia. *Nat. Neurosci.* **2015**, *18*, 631–636, doi:10.1038/nn.4000.

38. Cirulli, E.T.; Lasseigne, B.N.; Petrovski, S.; Sapp, P.C.; Dion, P.A.; Leblond, C.S.; Couthouis, J.; Lu, Y.-F.; Wang, Q.; Krueger, B.J.; et al. Exome sequencing in amyotrophic lateral sclerosis identifies risk genes and pathways. *Science (80-. ).* **2015**, *347*, 1436–1441, doi:10.1126/science.aaa3650.

39. Williams, K.L.; Topp, S.; Yang, S.; Smith, B.; Fifita, J.A.; Warraich, S.T.; Zhang, K.Y.; Farrawell, N.; Vance, C.; Hu, X.; et al. CCNF mutations in amyotrophic lateral sclerosis and frontotemporal dementia. *Nat. Commun.* **2016**, *7*, 11253, doi:10.1038/ncomms11253.

40. Spataro, R.; Kousi, M.; Farhan, S.M.K.; Willer, J.R.; Ross, J.P.; Dion, P.A.; Rouleau, G.A.; Daly, M.J.; Neale, B.M.; La Bella, V.; et al. Mutations in ATP13A2 (PARK9) are associated with an amyotrophic lateral sclerosis-like phenotype, implicating this locus in further phenotypic expansion. *Hum. Genomics* **2019**, *13*, 19, doi:10.1186/s40246-019-0203-9.

41. Yun, Y.; Hong, S.-A.; Kim, K.-K.; Baek, D.; Lee, D.; Londhe, A.M.; Lee, M.; Yu, J.; McEachin, Z.T.; Bassell, G.J.; et al. CRISPR-mediated gene correction links the ATP7A M1311V mutations with amyotrophic lateral sclerosis pathogenesis in one individual. *Commun. Biol.* **2020**, *3*, 33, doi:10.1038/s42003-020-0755-1.

42. van Rheenen, W.; Shatunov, A.; Dekker, A.M.; McLaughlin, R.L.; Diekstra, F.P.; Pulit, S.L.; van der Spek, R.A.A.; Võsa, U.; de Jong, S.; Robinson, M.R.; et al. Genome-wide association analyses identify new risk variants and the genetic architecture of amyotrophic lateral sclerosis. *Nat. Genet.* **2016**, *48*, 1043–1048, doi:10.1038/ng.3622.

43. Lee, T.; Li, Y.R.; Chesi, A.; Hart, M.P.; Ramos, D.; Jethava, N.; Hosangadi, D.; Epstein, J.; Hodges, B.; Bonini, N.M.; et al. Evaluating the prevalence of polyglutamine repeat expansions in amyotrophic lateral sclerosis. *Neurology* **2011**, *76*, 2062–2065, doi:10.1212/WNL.0b013e31821f4447.

44. Brenner, D.; Müller, K.; Gastl, R.; Gorges, M.; Otto, M.; Pinkhardt, E.H.; Kassubek, J.; Weishaupt, J.H.; Ludolph, A.C. Analysis of CACNA1A CAG repeat lengths in patients with familial ALS. *Neurobiol. Aging* **2019**, *74*, 235.e5-235.e8, doi:10.1016/j.neurobiolaging.2018.09.019.

45. Dobson-Stone, C.; Hallupp, M.; Shahheydari, H.; Ragagnin, A.M.G.; Chatterton, Z.; Carew-Jones, F.; Shepherd, C.E.; Stefen, H.; Paric, E.; Fath, T.; et al. CYLD is a causative gene for frontotemporal dementia – amyotrophic lateral sclerosis. *Brain* **2020**, *143*, 783–799, doi:10.1093/brain/awaa039.

46. Mitchell, J.; Paul, P.; Chen, H.-J.; Morris, A.; Payling, M.; Falchi, M.; Habgood, J.; Panoutsou, S.; Winkler, S.; Tisato, V.; et al. Familial amyotrophic lateral sclerosis is associated with a mutation in D-amino acid oxidase. *Proc. Natl. Acad. Sci.* **2010**, *107*, 7556–7561, doi:10.1073/pnas.0914128107.

47. Puls, I.; Jonnakuty, C.; LaMonte, B.H.; Holzbaur, E.L.F.; Tokito, M.; Mann, E.; Floeter, M.K.; Bidus, K.; Drayna, D.; Oh, S.J.; et al. Mutant dynactin in motor neuron disease. *Nat. Genet.* **2003**, *33*, 455–456, doi:10.1038/ng1123.

48. Farhan, S.M.K.; Howrigan, D.P.; Abbott, L.E.; Klim, J.R.; Topp, S.D.; Byrnes, A.E.; Churchhouse, C.; Phatnani, H.; Smith, B.N.; Rampersaud, E.; et al. Exome sequencing in amyotrophic lateral sclerosis implicates a novel gene, DNAJC7, encoding a heat-shock protein. *Nat. Neurosci.* **2019**, *22*, 1966–1974, doi:10.1038/s41593-019-0530-0.

49. Tunca, C.; Akçimen, F.; Coşkun, C.; Gündoğdu-Eken, A.; Kocoglu, C.; Çevik, B.; Bekircan-Kurt, C.E.; Tan, E.; Başak, A.N. ERLIN1 mutations cause teenage-onset slowly progressive ALS in a large Turkish pedigree. *Eur. J. Hum. Genet.* **2018**, *26*, 745–748, doi:10.1038/s41431-018-0107-5.

50. Amador, M.-D.-M.; Muratet, F.; Teyssou, E.; Banneau, G.; Danel-Brunaud, V.; Allart, E.; Antoine, J.-C.; Camdessanché, J.-P.; Anheim, M.; Rudolf, G.; et al. Spastic paraplegia due to recessive or dominant mutations in *ERLIN2* can convert to ALS. *Neurol. Genet.* **2019**, *5*, e374, doi:10.1212/NXG.0000000000000374.

51. Couthouis, J.; Hart, M.P.; Erion, R.; King, O.D.; Diaz, Z.; Nakaya, T.; Ibrahim, F.; Kim, H.-J.; Mojsilovic-Petrovic, J.; Panossian, S.; et al. Evaluating the role of the FUS/TLS-related gene EWSR1 in amyotrophic lateral sclerosis. *Hum. Mol. Genet.* **2012**, *21*, 2899–2911, doi:10.1093/hmg/dds116.

52. Corcia, P.; Brulard, C.; Beltran, S.; Marouillat, S.; Bakkouche, S.E.; Andres, C.R.; Blasco, H.; Vourc’h, P. Typical bulbar ALS can be linked to GARS mutation. *Amyotroph. Lateral Scler. Front. Degener.* **2019**, *20*, 275–277, doi:10.1080/21678421.2018.1556699.

53. Banks, G.T.; Bros-Facer, V.; Williams, H.P.; Chia, R.; Achilli, F.; Bryson, J.B.; Greensmith, L.; Fisher, E.M.C. Mutant Glycyl-tRNA Synthetase (Gars) Ameliorates SOD1G93A Motor Neuron Degeneration Phenotype but Has Little Affect on Loa Dynein Heavy Chain Mutant Mice. *PLoS One* **2009**, *4*, e6218, doi:10.1371/journal.pone.0006218.

54. Kaneb, H.M.; Folkmann, A.W.; Belzil, V. V.; Jao, L.-E.; Leblond, C.S.; Girard, S.L.; Daoud, H.; Noreau, A.; Rochefort, D.; Hince, P.; et al. Deleterious mutations in the essential mRNA metabolism factor, hGle1, in amyotrophic lateral sclerosis. *Hum. Mol. Genet.* **2015**, *24*, 1363–1373, doi:10.1093/hmg/ddu545.

55. Cooper-Knock, J.; Moll, T.; Ramesh, T.; Castelli, L.; Beer, A.; Robins, H.; Fox, I.; Niedermoser, I.; Van Damme, P.; Moisse, M.; et al. Mutations in the Glycosyltransferase Domain of GLT8D1 Are Associated with Familial Amyotrophic Lateral Sclerosis. *Cell Rep.* **2019**, *26*, 2298-2306.e5, doi:10.1016/j.celrep.2019.02.006.

56. Schymick, J.C.; Yang, Y.; Andersen, P.M.; Vonsattel, J.P.; Greenway, M.; Momeni, P.; Elder, J.; Chio, A.; Restagno, G.; Robberecht, W.; et al. Progranulin mutations and amyotrophic lateral sclerosis or amyotrophic lateral sclerosis-frontotemporal dementia phenotypes. *J. Neurol. Neurosurg. Psychiatry* **2006**, *78*, 754–756, doi:10.1136/jnnp.2006.109553.

57. Vance, C.; Al-Chalabi, A.; Ruddy, D.; Smith, B.N.; Hu, X.; Sreedharan, J.; Siddique, T.; Schelhaas, H.J.; Kusters, B.; Troost, D.; et al. Familial amyotrophic lateral sclerosis with frontotemporal dementia is linked to a locus on chromosome 9p13.2–21.3. *Brain* **2006**, *129*, 868–876, doi:10.1093/brain/awl030.

58. Figlewicz, D.A.; Krizus, A.; Martinoli, M.G.; Meininger, V.; Dib, M.; Rouleau, G.A.; Julien, J.-P. Variants of the heavy neurofilament subunit are associated with the development of amyotrophic lateral sclerosis. *Hum. Mol. Genet.* **1994**, *3*, 1757–1761, doi:10.1093/hmg/3.10.1757.

59. Al-Chalabi, A. Deletions of the heavy neurofilament subunit tail in amyotrophic lateral sclerosis. *Hum. Mol. Genet.* **1999**, *8*, 157–164, doi:10.1093/hmg/8.2.157.

60. Leung, C.L.; He, C.Z.; Kaufmann, P.; Chin, S.S.; Naini, A.; Liem, R.K.H.; Mitsumoto, H.; Hays, A.P. A Pathogenic Peripherin Gene Mutation in a Patient with Amyotrophic Lateral Sclerosis. *Brain Pathol.* **2006**, *14*, 290–296, doi:10.1111/j.1750-3639.2004.tb00066.x.

61. Heo, K.; Lim, S.M.; Nahm, M.; Kim, Y.-E.; Oh, K.-W.; Park, H.T.; Ki, C.-S.; Kim, S.H.; Lee, S. A *De Novo* *RAPGEF2* Variant Identified in a Sporadic Amyotrophic Lateral Sclerosis Patient Impairs Microtubule Stability and Axonal Mitochondria Distribution. *Exp. Neurobiol.* **2018**, *27*, 550–563, doi:10.5607/en.2018.27.6.550.

62. Meyer, T.; Schwan, A.; Dullinger, J.S.; Brocke, J.; Hoffmann, K.-T.; Nolte, C.H.; Hopt, A.; Kopp, U.; Andersen, P.; Epplen, J.T.; et al. Early-onset ALS with long-term survival associated with spastin gene mutation. *Neurology* **2005**, *65*, 141–143, doi:10.1212/01.wnl.0000167130.31618.0a.

63. Osmanovic, A.; Widjaja, M.; Förster, A.; Weder, J.; Wattjes, M.P.; Lange, I.; Sarikidi, A.; Auber, B.; Raab, P.; Christians, A.; et al. SPG7 mutations in amyotrophic lateral sclerosis: a genetic link to hereditary spastic paraplegia. *J. Neurol.* **2020**, doi:10.1007/s00415-020-09861-w.

64. Krüger, S.; Battke, F.; Sprecher, A.; Munz, M.; Synofzik, M.; Schöls, L.; Gasser, T.; Grehl, T.; Prudlo, J.; Biskup, S. Rare Variants in Neurodegeneration Associated Genes Revealed by Targeted Panel Sequencing in a German ALS Cohort. *Front. Mol. Neurosci.* **2016**, *9*, doi:10.3389/fnmol.2016.00092.

65. Chesi, A.; Staahl, B.T.; Jovičić, A.; Couthouis, J.; Fasolino, M.; Raphael, A.R.; Yamazaki, T.; Elias, L.; Polak, M.; Kelly, C.; et al. Exome sequencing to identify de novo mutations in sporadic ALS trios. *Nat. Neurosci.* **2013**, *16*, 851–855, doi:10.1038/nn.3412.

66. Ticozzi, N.; Vance, C.; LeClerc, A.L.; Keagle, P.; Glass, J.D.; McKenna-Yasek, D.; Sapp, P.C.; Silani, V.; Bosco, D.A.; Shaw, C.E.; et al. Mutational analysis reveals the FUS homolog TAF15 as a candidate gene for familial amyotrophic lateral sclerosis. *Am. J. Med. Genet. Part B Neuropsychiatr. Genet.* **2011**, *156*, 285–290, doi:10.1002/ajmg.b.31158.

67. Mackenzie, I.R.; Nicholson, A.M.; Sarkar, M.; Messing, J.; Purice, M.D.; Pottier, C.; Annu, K.; Baker, M.; Perkerson, R.B.; Kurti, A.; et al. TIA1 Mutations in Amyotrophic Lateral Sclerosis and Frontotemporal Dementia Promote Phase Separation and Alter Stress Granule Dynamics. *Neuron* **2017**, *95*, 808-816.e9, doi:10.1016/j.neuron.2017.07.025.

68. van der Spek, R.A.; van Rheenen, W.; Pulit, S.L.; Kenna, K.P.; Ticozzi, N.; Kooyman, M.; Mclaughlin, R.L.; Moisse, M.; van Eijk, K.R.; van Vugt, J.J.F.A.; et al. Reconsidering the causality of TIA1 mutations in ALS. *Amyotroph. Lateral Scler. Front. Degener.* **2018**, *19*, 1–3, doi:10.1080/21678421.2017.1413118.

69. Yamaura, G.; Higashiyama, Y.; Kusama, K.; Kunii, M.; Tanaka, K.; Koyano, S.; Nakashima, M.; Tsurusaki, Y.; Miyake, N.; Saitsu, H.; et al. Novel &lt;i&gt;VRK1&lt;/i&gt; Mutations in a Patient with Childhood-onset Motor Neuron Disease. *Intern. Med.* **2019**, *58*, 2715–2719, doi:10.2169/internalmedicine.2126-18.

70. Droppelmann, C.A.; Wang, J.; Campos-Melo, D.; Keller, B.; Volkening, K.; Hegele, R.A.; Strong, M.J. Detection of a novel frameshift mutation and regions with homozygosis within ARHGEF28 gene in familial amyotrophic lateral sclerosis. *Amyotroph. Lateral Scler. Front. Degener.* **2013**, *14*, 444–451, doi:10.3109/21678421.2012.758288.

71. Song, Y.; Lin, F.; Ye, C.; Huang, H.; Li, X.; Yao, X.; Xu, Y.; Wang, C. Rare, low-frequency and common coding variants of ARHGEF28 gene and their association with sporadic amyotrophic lateral sclerosis. *Neurobiol. Aging* **2020**, *87*, 138.e1-138.e6, doi:10.1016/j.neurobiolaging.2019.02.021.

72. Li, W.; Liu, Z.; Sun, W.; Yuan, Y.; Hu, Y.; Ni, J.; Jiao, B.; Fang, L.; Li, J.; Shen, L.; et al. Mutation analysis of GLT8D1 and ARPP21 genes in amyotrophic lateral sclerosis patients from mainland China. *Neurobiol. Aging* **2020**, *85*, 156.e1-156.e4, doi:10.1016/j.neurobiolaging.2019.09.013.

73. Alavi, A.; Malakouti Nejad, M.; Shahidi, G.; Elahi, E. Mutations in C19orf12 and intronic repeat expansions in C9orf72 not observed in Iranian Parkinson’s disease patients. *Neurobiol. Aging* **2017**, *54*, 214.e11-214.e12, doi:10.1016/j.neurobiolaging.2017.03.020.

74. Khani, M.; Alavi, A.; Shamshiri, H.; Zamani, B.; Hassanpour, H.; Kazemi, M.H.; Nafissi, S.; Elahi, E. Mutation screening of SLC52A3, C19orf12, and TARDBP in Iranian ALS patients. *Neurobiol. Aging* **2019**, *75*, 225.e9-225.e14, doi:10.1016/j.neurobiolaging.2018.11.003.

75. Deschauer, M.; Gaul, C.; Behrmann, C.; Prokisch, H.; Zierz, S.; Haack, T.B. C19orf12 mutations in neurodegeneration with brain iron accumulation mimicking juvenile amyotrophic lateral sclerosis. *J. Neurol.* **2012**, *259*, 2434–2439, doi:10.1007/s00415-012-6521-7.

76. Rzhepetskyy, Y.; Lazniewska, J.; Blesneac, I.; Pamphlett, R.; Weiss, N. *CACNA1H* missense mutations associated with amyotrophic lateral sclerosis alter Ca _v_ 3.2 T-type calcium channel activity and reticular thalamic neuron firing. *Channels* **2016**, *10*, 466–477, doi:10.1080/19336950.2016.1204497.

77. Gudesblatt, M.; Ludman, M.D.; Cohen, J.A.; Desnick, R.J.; Chester, S.; Grabowski, G.A.; Caroscio, J.T. Hexosaminidase a activity and amyotrophic lateral sclerosis. *Muscle Nerve* **1988**, *11*, 227–230, doi:10.1002/mus.880110307.

78. Drory, V.E.; Birnbaum, M.; Peleg, L.; Goldman, B.; Korczyn, A.D. Hexosaminidase A deficiency is an uncommon cause of a syndrome mimicking amyotrophic lateral sclerosis. *Muscle Nerve* **2003**, *28*, 109–112, doi:10.1002/mus.10371.

79. Daoud, H.; Valdmanis, P.N.; Gros-Louis, F.; Belzil, V.; Spiegelman, D.; Henrion, E.; Diallo, O.; Desjarlais, A.; Gauthier, J.; Camu, W.; et al. Resequencing of 29 Candidate Genes in Patients With Familial and Sporadic Amyotrophic Lateral Sclerosis. *Arch. Neurol.* **2011**, *68*, doi:10.1001/archneurol.2010.351.

80. Kim, H.; Lim, J.; Bao, H.; Jiao, B.; Canon, S.M.; Epstein, M.P.; Xu, K.; Jiang, J.; Parameswaran, J.; Li, Y.; et al. Rare variants in MYH15 modify amyotrophic lateral sclerosis risk. *Hum. Mol. Genet.* **2019**, *28*, 2309–2318, doi:10.1093/hmg/ddz063.

81. Jackson, M.; Morrison, K.E.; Al-Chalabi, A.; Baldier, M.; Leigh, P.N. Analysis of chromosome 5q13 genes in amyotrophic lateral sclerosis: Homozygous naip deletion in a sporadic case. *Ann. Neurol.* **1996**, *39*, 796–800, doi:10.1002/ana.410390616.

82. Kano, O.; Tanaka, K.; Kanno, T.; Iwasaki, Y.; Ikeda, J.-E. Neuronal apoptosis inhibitory protein is implicated in amyotrophic lateral sclerosis symptoms. *Sci. Rep.* **2018**, *8*, 6, doi:10.1038/s41598-017-18627-w.

83. Özoğuz, A.; Uyan, Ö.; Birdal, G.; Iskender, C.; Kartal, E.; Lahut, S.; Ömür, Ö.; Agim, Z.S.; Eken, A.G.; Sen, N.E.; et al. The distinct genetic pattern of ALS in Turkey and novel mutations. *Neurobiol. Aging* **2015**, *36*, 1764.e9-1764.e18, doi:10.1016/j.neurobiolaging.2014.12.032.

84. Rainier, S.; Bui, M.; Mark, E.; Thomas, D.; Tokarz, D.; Ming, L.; Delaney, C.; Richardson, R.J.; Albers, J.W.; Matsunami, N.; et al. Neuropathy Target Esterase Gene Mutations Cause Motor Neuron Disease. *Am. J. Hum. Genet.* **2008**, *82*, 780–785, doi:10.1016/j.ajhg.2007.12.018.

85. Kawarai, T.; Morita, M.; Morigaki, R.; Fujita, K.; Nodera, H.; Izumi, Y.; Goto, S.; Nakano, I.; Kaji, R. Pathomechanisms of motor neuron death by mutant TFG. *Rinsho Shinkeigaku* **2013**, *53*, 1199, doi:10.5692/clinicalneurol.53.1199.

86. Hermosura, M.C.; Nayakanti, H.; Dorovkov, M. V.; Calderon, F.R.; Ryazanov, A.G.; Haymer, D.S.; Garruto, R.M. A TRPM7 variant shows altered sensitivity to magnesium that may contribute to the pathogenesis of two Guamanian neurodegenerative disorders. *Proc. Natl. Acad. Sci.* **2005**, *102*, 11510–11515, doi:10.1073/pnas.0505149102.

87. Hara, K.; Kokubo, Y.; Ishiura, H.; Fukuda, Y.; Miyashita, A.; Kuwano, R.; Sasaki, R.; Goto, J.; Nishizawa, M.; Kuzuhara, S.; et al. *TRPM7* is not associated with amyotrophic lateral sclerosis-parkinsonism dementia complex in the Kii peninsula of Japan. *Am. J. Med. Genet. Part B Neuropsychiatr. Genet.* **2009**, *9999B*, n/a-n/a, doi:10.1002/ajmg.b.30966.

88. Kim, S.H.; Shi, Y.; Hanson, K.A.; Williams, L.M.; Sakasai, R.; Bowler, M.J.; Tibbetts, R.S. Potentiation of Amyotrophic Lateral Sclerosis (ALS)-associated TDP-43 Aggregation by the Proteasome-targeting Factor, Ubiquilin 1. *J. Biol. Chem.* **2009**, *284*, 8083–8092, doi:10.1074/jbc.M808064200.

89. González-Pérez, P.; Lu, Y.; Chian, R.-J.; Sapp, P.C.; Tanzi, R.E.; Bertram, L.; McKenna-Yasek, D.; Gao, F.-B.; Brown, R.H. Association of UBQLN1 mutation with Brown–Vialetto–Van Laere syndrome but not typical ALS. *Neurobiol. Dis.* **2012**, *48*, 391–398, doi:10.1016/j.nbd.2012.06.018.

90. Buscema, M.; Penco, S.; Grossi, E. A Novel Mathematical Approach to Define the Genes/SNPs Conferring Risk or Protection in Sporadic Amyotrophic Lateral Sclerosis Based on Auto Contractive Map Neural Networks and Graph Theory. *Neurol. Res. Int.* **2012**, *2012*, 1–13, doi:10.1155/2012/478560.

91. Kamel, F.; Umbach, D.M.; Lehman, T.A.; Park, L.P.; Munsat, T.L.; Shefner, J.M.; Sandler, D.P.; Hu, H.; Taylor, J.A. Amyotrophic lateral sclerosis, lead, and genetic susceptibility: polymorphisms in the delta-aminolevulinic acid dehydratase and vitamin D receptor genes. *Environ. Health Perspect.* **2003**, *111*, 1335–1339, doi:10.1289/ehp.6109.

92. Hayward, C.; Colville, S.; Swingler, R.J.; Brock, D.J.H. Molecular genetic analysis of the APEX nuclease gene in amyotrophic lateral sclerosis. *Neurology* **1999**, *52*, 1899–1899, doi:10.1212/WNL.52.9.1899.

93. Coppedè, F.; Gerfo, A. Lo; Carlesi, C.; Piazza, S.; Mancuso, M.; Pasquali, L.; Murri, L.; Migliore, L.; Siciliano, G. Lack of association between the APEX1 Asp148Glu polymorphism and sporadic amyotrophic lateral sclerosis. *Neurobiol. Aging* **2010**, *31*, 353–355, doi:10.1016/j.neurobiolaging.2008.03.018.

94. Zetterberg, H.; Jacobsson, J.; Rosengren, L.; Blennow, K.; Andersen, P.M. Association of APOE with age at onset of sporadic amyotrophic lateral sclerosis. *J. Neurol. Sci.* **2008**, *273*, 67–69, doi:10.1016/j.jns.2008.06.025.

95. Govone, F.; Vacca, A.; Rubino, E.; Gai, A.; Boschi, S.; Gentile, S.; Orsi, L.; Pinessi, L.; Rainero, I. Lack of association between APOE gene polymorphisms and amyotrophic lateral sclerosis: A comprehensive meta-analysis. *Amyotroph. Lateral Scler. Front. Degener.* **2014**, *15*, 551–556, doi:10.3109/21678421.2014.918149.

96. Conforti, F.L.; Spataro, R.; Sproviero, W.; Mazzei, R.; Cavalcanti, F.; Condino, F.; Simone, I.L.; Logroscino, G.; Patitucci, A.; Magariello, A.; et al. Ataxin-1 and ataxin-2 intermediate-length PolyQ expansions in amyotrophic lateral sclerosis. *Neurology* **2012**, *79*, 2315–2320, doi:10.1212/WNL.0b013e318278b618.

97. Lattante, S.; Pomponi, M.G.; Conte, A.; Marangi, G.; Bisogni, G.; Patanella, A.K.; Meleo, E.; Lunetta, C.; Riva, N.; Mosca, L.; et al. ATXN1 intermediate-length polyglutamine expansions are associated with amyotrophic lateral sclerosis. *Neurobiol. Aging* **2018**, *64*, 157.e1-157.e5, doi:10.1016/j.neurobiolaging.2017.11.011.

98. Elden, A.C.; Kim, H.-J.; Hart, M.P.; Chen-Plotkin, A.S.; Johnson, B.S.; Fang, X.; Armakola, M.; Geser, F.; Greene, R.; Lu, M.M.; et al. Ataxin-2 intermediate-length polyglutamine expansions are associated with increased risk for ALS. *Nature* **2010**, *466*, 1069–1075, doi:10.1038/nature09320.

99. Neuenschwander, A.G.; Thai, K.K.; Figueroa, K.P.; Pulst, S.M. Amyotrophic Lateral Sclerosis Risk for Spinocerebellar Ataxia Type 2 *ATXN2* CAG Repeat Alleles. *JAMA Neurol.* **2014**, *71*, 1529, doi:10.1001/jamaneurol.2014.2082.

100. van Blitterswijk, M.; Mullen, B.; Heckman, M.G.; Baker, M.C.; DeJesus-Hernandez, M.; Brown, P.H.; Murray, M.E.; Hsiung, G.-Y.R.; Stewart, H.; Karydas, A.M.; et al. Ataxin-2 as potential disease modifier in C9ORF72 expansion carriers. *Neurobiol. Aging* **2014**, *35*, 2421.e13-2421.e17, doi:10.1016/j.neurobiolaging.2014.04.016.

101. Tarr, I.S.; McCann, E.P.; Benyamin, B.; Peters, T.J.; Twine, N.A.; Zhang, K.Y.; Zhao, Q.; Zhang, Z.-H.; Rowe, D.B.; Nicholson, G.A.; et al. Monozygotic twins and triplets discordant for amyotrophic lateral sclerosis display differential methylation and gene expression. *Sci. Rep.* **2019**, *9*, 8254, doi:10.1038/s41598-019-44765-4.

102. Gros-Louis, F.; Andersen, P.M.; Dupre, N.; Urushitani, M.; Dion, P.; Souchon, F.; D’Amour, M.; Camu, W.; Meininger, V.; Bouchard, J.-P.; et al. Chromogranin B P413L variant as risk factor and modifier of disease onset for amyotrophic lateral sclerosis. *Proc. Natl. Acad. Sci.* **2009**, *106*, 21777–21782, doi:10.1073/pnas.0902174106.

103. Yang, X.; Li, S.; Xing, D.; Li, P.; Li, C.; Qi, L.; Xu, Y.; Ren, H. Lack of association between the P413L variant of *chromogranin B* and ALS risk or age at onset: a meta-analysis. *Amyotroph. Lateral Scler. Front. Degener.* **2018**, *19*, 80–86, doi:10.1080/21678421.2017.1361444.

104. Sabatelli, M.; Eusebi, F.; Al-Chalabi, A.; Conte, A.; Madia, F.; Luigetti, M.; Mancuso, I.; Limatola, C.; Trettel, F.; Sobrero, F.; et al. Rare missense variants of neuronal nicotinic acetylcholine receptor altering receptor function are associated with sporadic amyotrophic lateral sclerosis. *Hum. Mol. Genet.* **2009**, *18*, 3997–4006, doi:10.1093/hmg/ddp339.

105. Sabatelli, M.; Lattante, S.; Conte, A.; Marangi, G.; Luigetti, M.; Del Grande, A.; Chiò, A.; Corbo, M.; Giannini, F.; Mandrioli, J.; et al. Replication of association of CHRNA4 rare variants with sporadic amyotrophic lateral sclerosis: The Italian multicentre study. *Amyotroph. Lateral Scler.* **2012**, *13*, 580–584, doi:10.3109/17482968.2012.704926.

106. Xie, T.; Deng, L.; Mei, P.; Zhou, Y.; Wang, B.; Zhang, J.; Lin, J.; Wei, Y.; Zhang, X.; Xu, R. A genome-wide association study combining pathway analysis for typical sporadic amyotrophic lateral sclerosis in Chinese Han populations. *Neurobiol. Aging* **2014**, *35*, 1778.e9-1778.e23, doi:10.1016/j.neurobiolaging.2014.01.014.

107. Lopez-Lopez, A.; Gamez, J.; Syriani, E.; Morales, M.; Salvado, M.; Rodríguez, M.J.; Mahy, N.; Vidal-Taboada, J.M. CX3CR1 Is a Modifying Gene of Survival and Progression in Amyotrophic Lateral Sclerosis. *PLoS One* **2014**, *9*, e96528, doi:10.1371/journal.pone.0096528.

108. Siddons, M.A.; Pickering-Brown, S.M.; Mann, D.M.A.; Owen, F.; Cooper, P.N. Debrisoquine hydroxylase gene polymorphism frequencies in patients with amyotrophic lateral sclerosis. *Neurosci. Lett.* **1996**, *208*, 65–68, doi:10.1016/0304-3940(96)12549-0.

109. Deng, L.; Hou, L.; Zhang, J.; Tang, X.; Cheng, Z.; Li, G.; Fang, X.; Xu, J.; Zhang, X.; Xu, R. Polymorphism of rs3737597 in DISC1 Gene on Chromosome 1q42.2 in sALS Patients: a Chinese Han Population Case-Control Study. *Mol. Neurobiol.* **2017**, *54*, 3162–3179, doi:10.1007/s12035-016-9869-3.

110. van Es, M.A.; van Vught, P.W.; Blauw, H.M.; Franke, L.; Saris, C.G.; Van Den Bosch, L.; de Jong, S.W.; de Jong, V.; Baas, F.; van’t Slot, R.; et al. Genetic variation in DPP6 is associated with susceptibility to amyotrophic lateral sclerosis. *Nat. Genet.* **2008**, *40*, 29–31, doi:10.1038/ng.2007.52.

111. Fogh, I.; D’Alfonso, S.; Gellera, C.; Ratti, A.; Cereda, C.; Penco, S.; Corrado, L.; Sorarù, G.; Castellotti, B.; Tiloca, C.; et al. No association of DPP6 with amyotrophic lateral sclerosis in an Italian population. *Neurobiol. Aging* **2011**, *32*, 966–967, doi:10.1016/j.neurobiolaging.2009.05.014.

112. Blasco, H.; Bernard-Marissal, N.; Vourc’h, P.; Guettard, Y.O.; Sunyach, C.; Augereau, O.; Khederchah, J.; Mouzat, K.; Antar, C.; Gordon, P.H.; et al. A Rare Motor Neuron Deleterious Missense Mutation in the *DPYSL3* ( *CRMP4* ) Gene is Associated with ALS. *Hum. Mutat.* **2013**, *34*, 953–960, doi:10.1002/humu.22329.

113. Hafezparast, M. Mutations in Dynein Link Motor Neuron Degeneration to Defects in Retrograde Transport. *Science (80-. ).* **2003**, *300*, 808–812, doi:10.1126/science.1083129.

114. Shah, P.R.; Ahmad‐Annuar, A.; Ahmadi, K.R.; Russ, C.; Sapp, P.C.; Robert Horvitz, H.; Brown, R.H.; Goldstein, D.B.; Fisher, E.M.C. No association of *DYNC1H1* with sporadic ALS in a case‐control study of a northern European derived population: A tagging SNP approach. *Amyotroph. Lateral Scler.* **2006**, *7*, 46–56, doi:10.1080/14660820500397057.

115. Simpson, C.L.; Lemmens, R.; Miskiewicz, K.; Broom, W.J.; Hansen, V.K.; van Vught, P.W.J.; Landers, J.E.; Sapp, P.; Van Den Bosch, L.; Knight, J.; et al. Variants of the elongator protein 3 ( ELP3 ) gene are associated with motor neuron degeneration. *Hum. Mol. Genet.* **2009**, *18*, 472–481, doi:10.1093/hmg/ddn375.

116. Chiò, A.; Schymick, J.C.; Restagno, G.; Scholz, S.W.; Lombardo, F.; Lai, S.-L.; Mora, G.; Fung, H.-C.; Britton, A.; Arepalli, S.; et al. A two-stage genome-wide association study of sporadic amyotrophic lateral sclerosis. *Hum. Mol. Genet.* **2009**, *18*, 1524–1532, doi:10.1093/hmg/ddp059.

117. Van Es, M.A.; Van Vught, P.W.J.; Veldink, J.H.; Andersen, P.M.; Birve, A.; Lemmens, R.; Cronin, S.; Van Der Kooi, A.J.; De Visser, M.; Schelhaas, H.J.; et al. Analysis of *FGGY* as a risk factor for sporadic amyotrophic lateral sclerosis. *Amyotroph. Lateral Scler.* **2009**, *10*, 441–447, doi:10.3109/17482960802673042.

118. Goodall, E.F.; Greenway, M.J.; van Marion, I.; Carroll, C.B.; Hardiman, O.; Morrison, K.E. Association of the H63D polymorphism in the hemochromatosis gene with sporadic ALS. *Neurology* **2005**, *65*, 934–937, doi:10.1212/01.wnl.0000176032.94434.d4.

119. Yen, A.A.; Simpson, E.P.; Henkel, J.S.; Beers, D.R.; Appel, S.H. *HFE* mutations are not strongly associated with sporadic ALS. *Neurology* **2004**, *62*, 1611–1612, doi:10.1212/01.WNL.0000123114.04644.CC.

120. van Es, M.A.; Van Vught, P.W.; Blauw, H.M.; Franke, L.; Saris, C.G.; Andersen, P.M.; Van Den Bosch, L.; de Jong, S.W.; van ’t Slot, R.; Birve, A.; et al. ITPR2 as a susceptibility gene in sporadic amyotrophic lateral sclerosis: a genome-wide association study. *Lancet Neurol.* **2007**, *6*, 869–877, doi:10.1016/S1474-4422(07)70222-3.

121. Fernández-Santiago, R.; Sharma, M.; Berg, D.; Illig, T.; Anneser, J.; Meyer, T.; Ludolph, A.; Gasser, T. No evidence of association of FLJ10986 and ITPR2 with ALS in a large German cohort. *Neurobiol. Aging* **2011**, *32*, 551.e1-551.e4, doi:10.1016/j.neurobiolaging.2009.04.018.

122. Blauw, H.M.; van Rheenen, W.; Koppers, M.; Van Damme, P.; Waibel, S.; Lemmens, R.; van Vught, P.W.J.; Meyer, T.; Schulte, C.; Gasser, T.; et al. NIPA1 polyalanine repeat expansions are associated with amyotrophic lateral sclerosis. *Hum. Mol. Genet.* **2012**, *21*, 2497–2502, doi:10.1093/hmg/dds064.

123. Wills, A.-M.; Cronin, S.; Slowik, A.; Kasperaviciute, D.; Van Es, M.A.; Morahan, J.M.; Valdmanis, P.N.; Meininger, V.; Melki, J.; Shaw, C.E.; et al. A large-scale international meta-analysis of paraoxonase gene polymorphisms in sporadic ALS. *Neurology* **2009**, *73*, 16–24, doi:10.1212/WNL.0b013e3181a18674.

124. Panas, M.; Karadima, G.; Kalfakis, N.; Psarrou, O.; Floroskoufi, P.; Kladi, A.; Petersen, M.B.; Vassilopoulos, D. Genotyping of presenilin-1 polymorphism in amyotrophic lateral sclerosis. *J. Neurol.* **2000**, *247*, 940–942, doi:10.1007/s004150070050.

125. Saunderson, R.; Yu, B.; Trent, R.J.; Pamphlett, R. A polymorphism in the poliovirus receptor gene differs in motor neuron disease. *Neuroreport* **2004**, *15*, 383–386, doi:10.1097/00001756-200402090-00034.

126. Corcia, P.; Mayeux-Portas, V.; Khoris, J.; de Toffol, B.; Autret, A.; Müh, J.-P.; Camu, W.; Andres, C.; the French ALS Research Group Abnormal SMN1 gene copy number is a susceptibility factor for amyotrophic lateral sclerosis. *Ann. Neurol.* **2002**, *51*, 243–246, doi:10.1002/ana.10104.

127. Veldink, J.H.; van den Berg, L.H.; Cobben, J.M.; Stulp, R.P.; De Jong, J.M.B.V.; Vogels, O.J.; Baas, F.; Wokke, J.H.J.; Scheffer, H. Homozygous deletion of the survival motor neuron 2 gene is a prognostic factor in sporadic ALS. *Neurology* **2001**, *56*, 749–752, doi:10.1212/WNL.56.6.749.

128. Gamez, J.; Barcelo, M.J.; Munoz, X.; Carmona, F.; Cusco, I.; Baiget, M.; Cervera, C.; Tizzano, E.F. Survival and respiratory decline are not related to homozygous SMN2 deletions in ALS patients. *Neurology* **2002**, *59*, 1456–1460, doi:10.1212/01.WNL.0000032496.64510.4E.

129. Cady, J.; Koval, E.D.; Benitez, B.A.; Zaidman, C.; Jockel-Balsarotti, J.; Allred, P.; Baloh, R.H.; Ravits, J.; Simpson, E.; Appel, S.H.; et al. *TREM2* Variant p.R47H as a Risk Factor for Sporadic Amyotrophic Lateral Sclerosis. *JAMA Neurol.* **2014**, *71*, 449, doi:10.1001/jamaneurol.2013.6237.

130. van Es, M.A.; Veldink, J.H.; Saris, C.G.J.; Blauw, H.M.; van Vught, P.W.J.; Birve, A.; Lemmens, R.; Schelhaas, H.J.; Groen, E.J.N.; Huisman, M.H.B.; et al. Genome-wide association study identifies 19p13.3 (UNC13A) and 9p21.2 as susceptibility loci for sporadic amyotrophic lateral sclerosis. *Nat. Genet.* **2009**, *41*, 1083–1087, doi:10.1038/ng.442.

131. Lambrechts, D.; Poesen, K.; Fernandez-Santiago, R.; Al-Chalabi, A.; Del Bo, R.; Van Vught, P.W.J.; Khan, S.; Marklund, S.L.; Brockington, A.; van Marion, I.; et al. Meta-analysis of vascular endothelial growth factor variations in amyotrophic lateral sclerosis: increased susceptibility in male carriers of the -2578AA genotype. *J. Med. Genet.* **2009**, *46*, 840–846, doi:10.1136/jmg.2008.058222.

132. Iida, A.; Takahashi, A.; Kubo, M.; Saito, S.; Hosono, N.; Ohnishi, Y.; Kiyotani, K.; Mushiroda, T.; Nakajima, M.; Ozaki, K.; et al. A functional variant in ZNF512B is associated with susceptibility to amyotrophic lateral sclerosis in Japanese. *Hum. Mol. Genet.* **2011**, *20*, 3684–3692, doi:10.1093/hmg/ddr268.

133. Tetsuka, S.; Morita, M.; Iida, A.; Uehara, R.; Ikegawa, S.; Nakano, I. ZNF512B gene is a prognostic factor in patients with amyotrophic lateral sclerosis. *J. Neurol. Sci.* **2013**, *324*, 163–166, doi:10.1016/j.jns.2012.10.029.

134. Fogh, I.; Lin, K.; Tiloca, C.; Rooney, J.; Gellera, C.; Diekstra, F.P.; Ratti, A.; Shatunov, A.; van Es, M.A.; Proitsi, P.; et al. Association of a Locus in the *CAMTA1* Gene With Survival in Patients With Sporadic Amyotrophic Lateral Sclerosis. *JAMA Neurol.* **2016**, *73*, 812, doi:10.1001/jamaneurol.2016.1114.

135. Uyan, Ö.; Ömür, Ö.; Ağım, Z.S.; Özoğuz, A.; Li, H.; Parman, Y.; Deymeer, F.; Oflazer, P.; Koç, F.; Tan, E.; et al. Genome-Wide Copy Number Variation in Sporadic Amyotrophic Lateral Sclerosis in the Turkish Population: Deletion of EPHA3 Is a Possible Protective Factor. *PLoS One* **2013**, *8*, e72381, doi:10.1371/journal.pone.0072381.

136. Landers, J.E.; Melki, J.; Meininger, V.; Glass, J.D.; van den Berg, L.H.; van Es, M.A.; Sapp, P.C.; van Vught, P.W.J.; McKenna-Yasek, D.M.; Blauw, H.M.; et al. Reduced expression of the Kinesin-Associated Protein 3 (KIFAP3) gene increases survival in sporadic amyotrophic lateral sclerosis. *Proc. Natl. Acad. Sci.* **2009**, *106*, 9004–9009, doi:10.1073/pnas.0812937106.

137. Traynor, B.J.; Nalls, M.; Lai, S.-L.; Gibbs, R.J.; Schymick, J.C.; Arepalli, S.; Hernandez, D.; van der Brug, M.P.; Johnson, J.O.; Dillman, A.; et al. Kinesin-associated protein 3 (KIFAP3) has no effect on survival in a population-based cohort of ALS patients. *Proc. Natl. Acad. Sci.* **2010**, *107*, 12335–12338, doi:10.1073/pnas.0914079107.

138. Giess, R.; Beck, M.; Goetz, R.; Nitsch, R.M.; Toyka, K. V.; Sendtner, M. Potential role of LIF as a modifier gene in the pathogenesis of amyotrophic lateral sclerosis. *Neurology* **2000**, *54*, 1003–1005, doi:10.1212/WNL.54.4.1003.

139. Ringer, C.; Büning, L.-S.; Schäfer, M.K.H.; Eiden, L.E.; Weihe, E.; Schütz, B. PACAP signaling exerts opposing effects on neuroprotection and neuroinflammation during disease progression in the SOD1(G93A) mouse model of amyotrophic lateral sclerosis. *Neurobiol. Dis.* **2013**, *54*, 32–42, doi:10.1016/j.nbd.2013.02.010.

140. Kotni, M.K.; Zhao, M.; Wei, D.-Q. Gene expression profiles and protein-protein interaction networks in amyotrophic lateral sclerosis patients with C9orf72 mutation. *Orphanet J. Rare Dis.* **2016**, *11*, 148, doi:10.1186/s13023-016-0531-y.

141. Garofalo, O.; Figlewicz, D.A.; Leigh, P.N.; Powell, J.F.; Meininger, V.; Dib, M.; Rouleau, G.A. Androgen receptor gene polymorphisms in amyotrophic lateral sclerosis. *Neuromuscul. Disord.* **1993**, *3*, 195–199, doi:10.1016/0960-8966(93)90059-S.

142. Bruson, A.; Sambataro, F.; Querin, G.; D’Ascenzo, C.; Palmieri, A.; Agostini, J.; Gaiani, A.; Angelini, C.; Galbiati, M.; Poletti, A.; et al. CAG repeat length in androgen receptor gene is not associated with amyotrophic lateral sclerosis. *Eur. J. Neurol.* **2012**, *19*, 1373–1375, doi:10.1111/j.1468-1331.2011.03646.x.

143. Lennon, M.J.; Jones, S.P.; Lovelace, M.D.; Guillemin, G.J.; Brew, B.J. Bcl11b: A New Piece to the Complex Puzzle of Amyotrophic Lateral Sclerosis Neuropathogenesis? *Neurotox. Res.* **2016**, *29*, 201–207, doi:10.1007/s12640-015-9573-5.

144. Rothstein, J.D.; Dykes-Hoberg, M.; Corson, L.B.; Becker, M.; Cleveland, D.W.; Price, D.L.; Culotta, V.C.; Wong, P.C. The Copper Chaperone CCS Is Abundant in Neurons and Astrocytes in Human and Rodent Brain. *J. Neurochem.* **1999**, *72*, 422–429, doi:10.1046/j.1471-4159.1999.0720422.x.

145. Orrell, R.W.; King, A.W.; Lane, R.J.M.; de Belleroche, J.S. Investigation of a null mutation of the CNTF gene in familial amyotrophic lateral sclerosis. *J. Neurol. Sci.* **1995**, *132*, 126–128, doi:10.1016/0022-510X(95)00129-P.

146. Hommyo, R.; Suzuki, S.O.; Abolhassani, N.; Hamasaki, H.; Shijo, M.; Maeda, N.; Honda, H.; Nakabeppu, Y.; Iwaki, T. Expression of CRYM in different rat organs during development and its decreased expression in degenerating pyramidal tracts in amyotrophic lateral sclerosis. *Neuropathology* **2018**, *38*, 247–259, doi:10.1111/neup.12466.

147. Watanabe, M.; Jackson, M.; Ikeda, M.; Mizushima, K.; Amari, M.; Takatama, M.; Hirai, S.; Ikeda, Y.; Shizuka-Ikeda, M.; Okamoto, K. Genetic analysis of the cystatin C gene in familial and sporadic ALS patients. *Brain Res.* **2006**, *1073*–*1074*, 20–24, doi:10.1016/j.brainres.2005.12.046.

148. Van Hoecke, A.; Schoonaert, L.; Lemmens, R.; Timmers, M.; Staats, K.A.; Laird, A.S.; Peeters, E.; Philips, T.; Goris, A.; Dubois, B.; et al. EPHA4 is a disease modifier of amyotrophic lateral sclerosis in animal models and in humans. *Nat. Med.* **2012**, *18*, 1418–1422, doi:10.1038/nm.2901.

149. Capponi, S.; Geuens, T.; Geroldi, A.; Origone, P.; Verdiani, S.; Cichero, E.; Adriaenssens, E.; De Winter, V.; Bandettini di Poggio, M.; Barberis, M.; et al. Molecular Chaperones in the Pathogenesis of Amyotrophic Lateral Sclerosis: The Role of HSPB1. *Hum. Mutat.* **2016**, *37*, 1202–1208, doi:10.1002/humu.23062.

150. Vijayalakshmi, K.; Ostwal, P.; Sumitha, R.; Shruthi, S.; Varghese, A.M.; Mishra, P.; Manohari, S.G.; Sagar, B.C.; Sathyaprabha, T.N.; Nalini, A.; et al. Role of VEGF and VEGFR2 Receptor in Reversal of ALS-CSF Induced Degeneration of NSC-34 Motor Neuron Cell Line. *Mol. Neurobiol.* **2015**, *51*, 995–1007, doi:10.1007/s12035-014-8757-y.

151. Yao, R.-Q.; Ren, C.; Xia, Z.-F.; Yao, Y.-M. Organelle-specific autophagy in inflammatory diseases: a potential therapeutic target underlying the quality control of multiple organelles. *Autophagy* **2020**, 1–17, doi:10.1080/15548627.2020.1725377.

152. Li, P.-A.; He, Q.; Cao, T.; Yong, G.; Szauter, K.M.; Fong, K.S.K.; Karlsson, J.; Keep, M.F.; Csiszar, K. Up-regulation and altered distribution of lysyl oxidase in the central nervous system of mutant SOD1 transgenic mouse model of amyotrophic lateral sclerosis. *Mol. Brain Res.* **2004**, *120*, 115–122, doi:10.1016/j.molbrainres.2003.10.013.

153. OrrÃ^1^, S. Association of monoamine oxidase B alleles with age at onset in amyotrophic lateral sclerosis. *Neuromuscul. Disord.* **1999**, *9*, 593–597, doi:10.1016/S0960-8966(99)00052-8.

154. Wu, C.; Watts, M.E.; Rubin, L.L. MAP4K4 Activation Mediates Motor Neuron Degeneration in Amyotrophic Lateral Sclerosis. *Cell Rep.* **2019**, *26*, 1143-1156.e5, doi:10.1016/j.celrep.2019.01.019.

155. Watts, M.E.; Wu, C.; Rubin, L.L. Suppression of MAP4K4 Signaling Ameliorates Motor Neuron Degeneration in Amyotrophic Lateral Sclerosis-Molecular Studies Toward New Therapeutics. *J. Exp. Neurosci.* **2019**, *13*, 117906951986279, doi:10.1177/1179069519862798.

156. The microRNA miR-375-3p and the Tumor Suppressor NDRG2 are Involved in Sporadic Amyotrophic Lateral Sclerosis. *Cell. Physiol. Biochem.* **2019**, *52*, 1412–1426, doi:10.33594/000000099.

157. Murakami, T.; Nagai, M.; Miyazaki, K.; Morimoto, N.; Ohta, Y.; Kurata, T.; Takehisa, Y.; Kamiya, T.; Abe, K. Early decrease of mitochondrial DNA repair enzymes in spinal motor neurons of presymptomatic transgenic mice carrying a mutant SOD1 gene. *Brain Res.* **2007**, *1150*, 182–189, doi:10.1016/j.brainres.2007.02.057.

158. Yamashita, S.; Mori, A.; Kimura, E.; Mita, S.; Maeda, Y.; Hirano, T.; Uchino, M. DJ-1 forms complexes with mutant SOD1 and ameliorates its toxicity. *J. Neurochem.* **2010**, *113*, 860–870, doi:10.1111/j.1471-4159.2010.06658.x.

159. Recabarren-Leiva, D.; Alarcón, M. New insights into the gene expression associated to amyotrophic lateral sclerosis. *Life Sci.* **2018**, *193*, 110–123, doi:10.1016/j.lfs.2017.12.016.

160. Eschbach, J.; Schwalenstocker, B.; Soyal, S.M.; Bayer, H.; Wiesner, D.; Akimoto, C.; Nilsson, A.-C.; Birve, A.; Meyer, T.; Dupuis, L.; et al. PGC-1  is a male-specific disease modifier of human and experimental amyotrophic lateral sclerosis. *Hum. Mol. Genet.* **2013**, *22*, 3477–3484, doi:10.1093/hmg/ddt202.

161. Niwa, J.; Ishigaki, S.; Hishikawa, N.; Yamamoto, M.; Doyu, M.; Murata, S.; Tanaka, K.; Taniguchi, N.; Sobue, G. Dorfin Ubiquitylates Mutant SOD1 and Prevents Mutant SOD1-mediated Neurotoxicity. *J. Biol. Chem.* **2002**, *277*, 36793–36798, doi:10.1074/jbc.M206559200.

162. Vérièpe, J.; Fossouo, L.; Parker, J.A. Neurodegeneration in C. elegans models of ALS requires TIR-1/Sarm1 immune pathway activation in neurons. *Nat. Commun.* **2015**, *6*, 7319, doi:10.1038/ncomms8319.

163. Boutahar, N.; Wierinckx, A.; Camdessanche, J.P.; Antoine, J.-C.; Reynaud, E.; Lassabliere, F.; Lachuer, J.; Borg, J. Differential effect of oxidative or excitotoxic stress on the transcriptional profile of amyotrophic lateral sclerosis-linked mutant SOD1 cultured neurons. *J. Neurosci. Res.* **2011**, *89*, 1439–1450, doi:10.1002/jnr.22672.

164. Ikeda, J.; Kohriyama, T.; Nakamura, S. Elevation of serum soluble E-selectin and antisulfoglucuronyl paragloboside antibodies in amyotrophic lateral sclerosis. *Eur. J. Neurol.* **2000**, *7*, 541–547, doi:10.1046/j.1468-1331.2000.t01-1-00114.x.

165. Luisier, R.; Tyzack, G.E.; Hall, C.E.; Mitchell, J.S.; Devine, H.; Taha, D.M.; Malik, B.; Meyer, I.; Greensmith, L.; Newcombe, J.; et al. Intron retention and nuclear loss of SFPQ are molecular hallmarks of ALS. *Nat. Commun.* **2018**, *9*, 2010, doi:10.1038/s41467-018-04373-8.

166. Albani, D.; Pupillo, E.; Bianchi, E.; Chierchia, A.; Martines, R.; Forloni, G.; Beghi, E. The role of single-nucleotide variants of the energy metabolism-linked genes &lt;i&gt;SIRT3&lt;/i&gt;, &lt;i&gt;PPARGC1A&lt;/i&gt; and &lt;i&gt;APOE&lt;/i&gt; in amyotrophic lateral sclerosis risk. *Genes Genet. Syst.* **2016**, *91*, 301–309, doi:10.1266/ggs.16-00023.

167. Flowers, J.M.; Leigh, P.N.; Davies, A.M.; Ninkina, N.N.; Buchman, V.L.; Vaughan, J.; Wood, N.W.; Powell, J.F. Mutations in the gene encoding human persyn are not associated with amyotrophic lateral sclerosis or familial Parkinson’s disease. *Neurosci. Lett.* **1999**, *274*, 21–24, doi:10.1016/S0304-3940(99)00673-4.

168. Shibata, N.; Asayama, K.; Hirano, A.; Kobayashi, M. Immunohistochemical Study on Superoxide Dismutases in Spinal Cords from Autopsied Patients with Amyotrophic Lateral Sclerosis. *Dev. Neurosci.* **1996**, *18*, 492–498, doi:10.1159/000111445.

169. Jones, A.R.; Troakes, C.; King, A.; Sahni, V.; De Jong, S.; Bossers, K.; Papouli, E.; Mirza, M.; Al-Sarraj, S.; Shaw, C.E.; et al. Stratified gene expression analysis identifies major amyotrophic lateral sclerosis genes. *Neurobiol. Aging* **2015**, *36*, 2006.e1-2006.e9, doi:10.1016/j.neurobiolaging.2015.02.017.

170. Meisler, M.H.; Russ, C.; Montgomery, K.T.; Greenway, M.; Ennis, S.; Hardiman, O.; Figlewicz, D.A.; Quenneville, N.R.; Conibear, E.; Brown, R.H. Evaluation of the Golgi trafficking protein VPS54 ( *wobbler* ) as a candidate for ALS. *Amyotroph. Lateral Scler.* **2008**, *9*, 141–148, doi:10.1080/17482960801934403.

171. Schmitt-John, T.; Drepper, C.; Mußmann, A.; Hahn, P.; Kuhlmann, M.; Thiel, C.; Hafner, M.; Lengeling, A.; Heimann, P.; Jones, J.M.; et al. Mutation of Vps54 causes motor neuron disease and defective spermiogenesis in the wobbler mouse. *Nat. Genet.* **2005**, *37*, 1213–1215, doi:10.1038/ng1661.

172. Karczewski, K.J.; Francioli, L.C.; Tiao, G.; Cummings, B.B.; Alföldi, J.; Wang, Q.; Collins, R.L.; Laricchia, K.M.; Ganna, A.; Birnbaum, D.P.; et al. The mutational constraint spectrum quantified from variation in 141,456 humans. *Nature* **2020**, *581*, 434–443, doi:10.1038/s41586-020-2308-7.
